# Association of meat, vegetarian, pescatarian and fish-poultry diets with risk of 19 cancer sites and all cancer: findings from the UK Biobank prospective cohort study and meta-analysis

**DOI:** 10.1186/s12916-022-02257-9

**Published:** 2022-02-24

**Authors:** Solange Parra-Soto, Danay Ahumada, Fanny Petermann-Rocha, Jirapitcha Boonpoor, Jose Lara Gallegos, Jana Anderson, Linda Sharp, Fiona C. Malcomson, Katherine M. Livingstone, John C. Mathers, Jill P. Pell, Frederick K. Ho, Carlos Celis-Morales

**Affiliations:** 1 Institute of Health and Wellbeing, University of Glasgow, Glasgow, G12 8RZ UK; 2 British Heart Foundation Cardiovascular Research Centre, Institute of Cardiovascular and Medical Sciences, University of Glasgow, Glasgow, G12 8TA UK; 3 Department of Process and Evaluation, Faculty of Health and Life Sciences, Universidad Católica de Temuco, Temuco, Chile; 4 Facultad de Medicina, Universidad Diego Portales, Santiago, Chile; 5 Department of Applied Sciences, Faculty of Health and Life Sciences, Northumbria University, EBD223 Ellison Building, Newcastle upon Tyne, NE1 8ST UK; 6 Newcastle University Centre for Cancer, Population Health Sciences Institute, Newcastle University, Newcastle upon Tyne, UK; 7 Human Nutrition Research Centre, Centre for Healthier Lives, Population Health Sciences Institute, Newcastle University, Newcastle upon Tyne, NE2 4HH UK; 8 Institute for Physical Activity and Nutrition, School of Exercise and Nutrition Sciences, Deakin University, Geelong, VIC 3220 Australia; 9 Education, Physical Activity and Health Research Unit, University Católica del Maule, 3466706 Talca, Chile

**Keywords:** Cancer, Diet, Vegetarians, Pescatarian

## Abstract

**Background:**

The associations of cancer with types of diets, including vegetarian, fish, and poultry-containing diets, remain unclear. The aim of this study was, therefore, to investigate the association of type of diet with all cancers and 19 site-specific incident cancers in a prospective cohort study and then in a meta-analysis of published prospective cohort studies.

**Methods:**

A total of 409,110 participants from the UK Biobank study, recruited between 2006 and 2010, were included. The outcomes were incidence of all cancers combined and 19 cancer sites. Associations between the types of diets and cancer were investigated using Cox proportional hazards models. Previously published prospective cohort studies were identified from four databases, and a meta-analysis was conducted using random-effects models.

**Results:**

The mean follow-up period was 10.6 years (IQR 10.0; 11.3). Compared with meat-eaters, vegetarians (hazard ratio (HR) 0.87 [95% CI: 0.79 to 0.96]) and pescatarians (HR 0.93 [95% CI: 0.87 to 1.00]) had lower overall cancer risk. Vegetarians also had a lower risk of colorectal and prostate cancers compared with meat-eaters. In the meta-analysis, vegetarians (Risk Ratio (RR): 0.90 [0.86 to 0.94]) and pescatarians (RR 0.91 [0.86; 0.96]) had lower risk of overall and colorectal cancer. No associations between the types of diets and prostate, breast, or lung cancers were found.

**Conclusions:**

Compared with meat-eaters, vegetarians and pescatarians had a lower risk of overall, colorectal, and prostate cancer. When results were pooled in a meta-analysis, the associations with overall and colorectal cancer persisted, but the results relating to other specific cancer sites were inconclusive.

**Supplementary Information:**

The online version contains supplementary material available at 10.1186/s12916-022-02257-9.

## Introduction

Unhealthy diets have been associated with a higher risk of several adverse health outcomes, including cancer [1, 2]. Over 11 million deaths were attributed to poor diet in 2017, among which more than 930,000 were attributed to cancer, particularly, breast and colorectal cancer [1]. Although there is considerable evidence regarding the associations between diet and cancer risk, most studies have focused on single nutrients [3, 4] or food items [3, 5], which do not provide insights into how the widely varying combinations of these foods within whole dietary patterns impact risk.

What we eat and the type of dietary pattern we follow are influenced by socioeconomic status, environmental factors, and cultural and personal beliefs [1]. Recently, there has been a growing concern about the impact of food consumption on not just human health but also planetary health. This has led to an increasing number of people worldwide changing from diets that include meat to other types of diet such as vegetarian, vegan, and pescatarian [6–9]. Recent estimates indicate that 4% of the worldwide population are vegetarian, with almost 40% reporting frequent consumption of vegetarian meals [10]. Although there is increasing evidence regarding the health benefits associated with these types of diets, most studies have focused on outcomes such as all-cause mortality and cardiovascular diseases, with limited and conflicting evidence for all cancers combined and specific cancers [11–14]. One study conducted in 73,308 Seventh-day Adventist men and women who were followed up for 5.7 years reported a 12% lower risk of all-cause mortality in vegetarians compared with non-vegetarians [14]. Although no associations were observed for the incidence of all cancer combined or mortality [14], vegetarians had a lower risk of cancers of the gastrointestinal tract [15]. Another study that pooled data from two prospective cohorts, covering 61,647 British men and women who were followed for 14.9 years, reported that fish-eaters had 38%, 34% and 12% lower risk of stomach, colorectal, and all cancers, respectively [13], while vegetarians had 63% and 12% lower risk of stomach and all cancers, respectively, compared with meat-eaters [13]. A combined analysis of EPIC-Oxford and the Oxford Vegetarian Study, also from the UK, reported that vegetarians (including vegans) had a lower risk of all cancers and cancers of the stomach, bladder, lymphatic and haematopoietic system but a higher risk of cervical cancer compared with meat-eaters [16]. In the UK Women’s Cohort Study, a vegetarian diet was not associated with differences in the risk of breast cancer [17]. The latest meta-analysis for overall cancer, published in 2017, pooled only two prospective cohort studies and showed an 8% reduction in overall incident cancers associated with vegetarian diets [18]. Another meta-analysis, conducted in 2017, included nine studies (*n* = 686,629 participants) and reported no associations between a vegetarian diet and the risk of breast, colorectal or prostate cancers [19].

In summary, existing evidence shows that investigations of associations with dietary patterns have been restricted to a limited number of cancer sites with equivocal findings. We addressed these research gaps using data from the UK Biobank, a large prospective cohort study, to investigate the associations between the type of diet and all incident cancers combined and 19 site-specific cancers. In addition, we combined our findings with those from past studies to provide an up-to-date meta-analysis of prospective cohort studies.

## Methods

### UK Biobank

Between April 2007 and December 2010, the UK Biobank recruited over 500,000 participants, aged 37 to 73 years from the general population [20]. Participants attended one of 22 assessment centres across England, Wales and Scotland [21, 22], where they completed a touchscreen questionnaire, had physical measurements taken and provided biological samples, as described in detail elsewhere [21, 22].

### Outcomes

The primary outcome was incident cancer defined as the first record of hospitalization for cancer or death due to cancer, if no prior record of hospitalization. Date and cause of death were obtained from death certificates available up to 1 June 2020. Dates and causes of hospital admissions were obtained from the Health Episode Statistics (England and Wales) and Scottish Morbidity Records (Scotland). Follow-up for incident events was censored on this date or the date of the event (cancer diagnosis or death), whichever came first. The International Classification of Diseases, 10th revision (ICD-10) was used to define the overall cancer (C00–C97, excluding non-melanoma skin cancer (C44)) and the following 19 cancers and four subgroups of colorectal cancer: head and neck (C00–C14), oesophagus (C15), stomach (C16), colorectal (C18, C19 and C20), colon (C18.0), colon proximal (C18.0–18.4), colon distal (C18.5, C18.7), rectum (C19–C20), pancreas (C25), lung (C33–34), malignant melanoma (C43), breast in premenopausal and postmenopausal women (C50), uterus (C54–C55), ovary (C56), prostate (C61), kidney (C64–C65), bladder (C67), brain (C70–72), lymphatic and haematopoietic tissue (C81–C96), non-Hodgkin lymphoma (C82–C86, C96), multiple myeloma (C88–C90) and leukaemia (C91–C95). Further details of these measurements can be found in the UK Biobank online protocol [23].

### Types of diets

A touchscreen dietary intake questionnaire, containing 29 questions about diet and 18 questions about alcohol, completed at recruitment (baseline), was used to collect data on the frequency of food intake over the past year. Participants chose a frequency of intake ranging from “never” to “once or more daily” for each food item. The food items included were cheese, milk, fish (oily and non-oily), poultry and red meat (processed meat, beef, lamb or mutton, pork, chicken, turkey or other poultry). Participants were also asked to report whether they followed any particular diet, including gluten-free, lactose-free, low calorie, vegetarian and vegan diets. Based on their responses, participants were categorized into one of the following diets: vegetarian, which included lacto-ovo-vegetarian (who consumed cheese and/or milk but they never consumed fish, poultry or red meat) and vegan (who reported never consuming milk, cheese, fish, poultry or red meat); pescatarian (who consumed cheese, milk and fish but never consumed poultry or red meat); fish-poultry eaters (who consumed cheese, milk, fish and poultry but never consumed red meat); and meat-eaters (who consumed cheese, milk, fish, poultry and red meat). Due to the low number of participants following a vegan diet (*n* = 57), these were pooled with vegetarians. To take account of people changing their dietary patterns, we excluded people who self-reported at baseline that their diet often varied (*n* = 45,028, 8.99%). In addition, those participants who reported being vegetarians but who self-reported eating any meat products were excluded from the study (*n* = 57). The same approach was used for pescatarians and fish-poultry eaters [24]. Dietary information for total energy and macro- and micro-nutrients was collected via the Oxford WebQ, a web-based 24-hour dietary questionnaire [25]. Bradbury et al. reported that data collected using the dietary touchscreen questionnaire, which was applied to the entire cohort, correctly ranked subjects according to their primary food group intakes [26].

### Covariates

Sociodemographic factors (sex and ethnicity) were self-reported at the baseline assessment visit using a touchscreen questionnaire. Age was calculated from the date of birth at baseline assessment. Area-based socioeconomic status was derived from the postcode of residence using the Townsend score (16), which generates a deprivation score based on four census variables: unemployment, non-car ownership, non-house ownership and household overcrowding. Self-reported smoking status was categorized as never, former or current smoker. Body mass index (BMI) was calculated from weight and height expressed in kg/m^2^, and the World Health Organization (WHO) criteria were applied to classify participants into underweight (< 18.5 kg/m^2^), normal weight (18.5–24.9 kg/m^2^), overweight (25.0–29.9 kg/m^2^) and obese (≥ 30.0 kg/m^2^) [17]. Data were also collected from women on hormonal replacement therapy, menopausal status and parity. Self-reported levels of physical activity were collected via the International Physical Activity Questionnaire and reported as metabolic equivalent of task (MET) per week [27]. Multimorbidity (physician diagnosis of depression, hypertension and diabetes) was self-reported at baseline. For this study, an average of up to five 24-h recalls was used. However, as the average of the 24-h recalls was not available for the whole UK Biobank population (~ 200,000 individuals), the characteristics of individuals with data available for 24-hrs dietary recall is shown in Additional file 1: Table S2.

### Statistical analyses

We excluded people who had cancer diagnoses at baseline and people with missing data for all covariates studied and for the exposure of interest. Descriptive characteristics for the cohort, categorized by type of diet, were summarized using the means with standard deviations (SD) for quantitative variables and percentages for categorical variables.

Associations between the types of diet and all cancer combined, and the individual cancer sites were investigated using Cox-proportional hazard models. Individuals who were classified as meat-eaters were used as the reference group. The time of follow-up was used as the time-dependent variable. The results were reported as hazard ratios (HR) and their 95% confidence intervals (95% CI). The proportional hazard assumptions were checked using Schoenfeld residuals.

We ran four models for each outcome, including an increasing number of covariates: “model 0” unadjusted, “model 1” (minimally adjusted) included sociodemographic covariates (age, sex, deprivation index and ethnicity), “model 2” additionally included lifestyle factors (smoking, alcohol intake and total physical activity), “model 3” additionally included multimorbidity and “model 4” (maximally adjusted) additionally included BMI. The models for breast, ovarian, cervical, endometrial and uterine cancer were also adjusted for hormone replacement therapy and parity. To minimize the effect of reverse causation, we additionally conducted 2-year landmark sensitivity analyses, excluding cancer events in the first 2 years of follow-up. All analyses were undertaken using the R statistical software, version 3.6.2, with the package “survival”. Two-sided *P*-values below 0.05 were interpreted as statistically significant.

### Meta-analysis

The systematic review was conducted according to the Preferred Reporting Items for Systematic Review and Meta-Analyses (PRISMA) guidelines [28] and registered in PROSPERO with the number CRD42021240456. The research question was “Do vegetarians, vegan, fish- and poultry-eaters have a lower overall and site-specific cancer risk compared to meat-eaters?”. The population included was adults aged ≥ 18 years with and without a cancer diagnosis. The exposure was types of diet, including vegan, vegetarian, poultry eaters, fish eaters and meat-eaters. Outcomes included all cause- and site-specific (colorectal, breast, prostate, digestive tract and lung) cancers. Only prospective cohort studies were included. As recorded in PROSPERO, two authors (SP-S and DA) searched MEDLINE/PubMed, Scopus, Embase and Web of Science using the search terms described in the Additional file 1: Methods. Two stages of screening (1) the title and abstract and (2) the full text of potentially eligible papers were performed. Data extraction was carried out independently by the authors SP-S and DA in Rayyan, and the results were then extracted to an Excel spreadsheet. The inclusion was restricted to prospective cohort studies published any time up to and including 31 August 2021, which were conducted in adults, included some/all of the following types of diets (meat, vegetarian, pescatarian, fish and poultry diet), provided results for some/all of the relevant cancer outcomes and was written in English. We excluded case-control studies and studies that did not define the type of diet. Meta-analysis was undertaken using a random-effects model, stratified by type of diet (vegetarian, pescatarian or both) and only included specific cancer sites that were reported by at least three independent studies. Manuscripts that met the inclusion criteria were assessed independently by the two authors using the RAYYAN software [29]. We used funnel plots to assess the potential bias within the studies included in the meta-analyses. The quality of the studies was also assessed using the Newcastle-Ottawa Scale (Additional file 1: Table S1) [30]. Heterogeneity between studies was tested using the *I*^2^ statistic. All analyses were undertaken using the R statistical software, version 3.6.2 with the package “meta”.

## Results

### UK Biobank

Of the 502,492 UK participants, 99,382 were excluded as they had cancer diagnoses at baseline (*n* = 41,406) and missing data for types of diet or relevant covariates (*n* = 57,976) (baseline characteristics of people with missing variables are in Additional file 1: Table S2). Therefore, this study included 409,110 participants, of whom 53.4% were women. Overall, 7256 (1.8%) were vegetarian, 9498 (2.3%) were pescatarian, 4625 (1.1%) were fish-poultry eaters and 387,731 (94.8%) were meat-eaters (Additional file 1: Fig. S1). The median follow-up period was 10.6 years (interquartile range 10.0 to 11.3), and 39,596 participants developed cancers during follow-up. The sociodemographic characteristics of the population by type of diet are presented in Table 1. In comparison with the other groups, meat-eaters were older and more likely to be overweight or obese and to smoke (Table 1). The characteristics of dietary intake across types of diets are presented in Additional file 1: Table S2. Energy consumption was similar in the diet groups. However, intake of fibre, polyunsaturated fat and water was slightly higher in participants with vegetarian and pescatarian diets. In contrast, protein intake was lower in vegetarians (mean 12.4 ± 2.3 SD % of total energy) than in meat-eaters (mean 15.7 ± 3.6 SD % of total energy); crisps and pizza were more consumed by vegetarian and pescatarian. Additional file 1: Table S3 shows the characteristics of the UK Biobank population excluded from the study (*n* = 57,976). Briefly, compared with the cohort included in the present study, those excluded were more likely to be women, individuals from more deprived backgrounds, of non-white ethnicity and have a higher BMI.

**Table 1:** Sociodemographic characteristics of the study population by types of diet

Characteristics	Meat-eaters	Vegan and vegetarian	Pescatarian	Fish and poultry	Overall
**Sociodemographic**					
*N* (%)	387,731 (94.8%)	7256 (1.8%)	9498 (2.3%)	4625 (1.1%)	409,110
Age, mean (SD)	56.4 (8.09)	52.9 (7.92)	53.9 (8.03)	56.2 (8.12)	56.3 (8.11)
Sex, *n* (%)					
Females	203,550 (52.5%)	4770 (65.7%)	6770 (71.3%)	3477 (75.2%)	218,567 (53.4%)
Males	184,181 (47.5%)	2486 (34.3%)	2728 (28.7%)	1148 (24.8%)	190,543 (46.6%)
Deprivation, *n* (%)					
Lower	133,296 (34.4%)	1882 (25.9%)	2766 (29.1%)	1300 (28.1%)	139,244 (34.0%)
Middle	130,632 (33.7%)	2337 (32.2%)	3155 (33.2%)	1466 (31.7%)	137,590 (33.6%)
Higher	123,803 (31.9%)	3037 (41.9%)	3577 (37.7%)	1859 (40.2%)	132,276 (32.3%)
Ethnicity, *n* (%)					
White	368,509 (95.0%)	5825 (80.3%)	8882 (93.5%)	4150 (89.7%)	387,366 (94.7%)
Mixed	5309 (1.4%)	116 (1.6%)	142 (1.5%)	96 (2.1%)	5663 (1.4%)
South Asian	5776 (1.5%)	1235 (17.0%)	284 (3.0%)	217 (4.7%)	7512 (1.8%)
Black	5775 (1.5%)	30 (0.4%)	131 (1.4%)	143 (3.1%)	6079 (1.5%)
Chinese	1206 (0.3%)	9 (0.1%)	10 (0.1%)	5 (0.1%)	1230 (0.3%)
Any other	1156 (0.3%)	41 (0.6%)	49 (0.5%)	14 (0.3%)	1260 (0.3%)
**Anthropometric**					
Height (m), mean (SD)	1.68 (0.09)	1.66 (0.09)	1.67 (0.09)	1.65 (0.09)	1.68 (0.09)
Weight (kg), mean (SD)	78.4 (15.81)	71.5 (14.66)	70.7 (13.82)	70.3 (14.15)	78.0 (15.82)
Waist (cm), mean (SD)	90.4 (13.35)	85.1 (12.80)	83.2 (12.07)	83.3 (12.57)	90.1 (13.38)
Body mass index (kg/m^2^), mean (SD)	27.4 (4.71)	25.7 (4.63)	25.2 (4.24)	25.6 (4.60)	27.3 (4.72)
BMI (kg/m^2^), *n* (%)					
Underweight	1752 (0.5%)	118 (1.6%)	155 (1.6%)	71 (1.5%)	2096 (0.5%)
Normal	124,548 (32.1%)	3556 (49.0%)	5010 (52.7%)	2285 (49.4%)	135,399 (33.1%)
Overweight	167,563 (43.2%)	2531 (34.9%)	3215 (33.8%)	1572 (34.0%)	174,881 (42.7%)
Obese	93,868 (24.2%)	1051 (14.5%)	1118 (11.8%)	697 (15.1%)	96,734 (23.6%)
**Lifestyle**					
Smoking, *n* (%)					
Never	214,263 (55.3%)	4676 (64.4%)	5437 (57.2%)	2763 (59.7%)	227,139 (55.5%)
Previous	133,411 (34.4%)	2086 (28.7%)	3390 (35.7%)	1511 (32.7%)	140,398 (34.3%)
Current	40,057 (10.3%)	494 (6.8%)	671 (7.1%)	351 (7.6%)	41,573 (10.2%)
Alcohol intake, *n* (%)					
Daily or almost daily	79,854 (20.6%)	1015 (14.0%)	1825 (19.2%)	641 (13.9%)	83,335 (20.4%)
3-4 times a week	91,813 (23.7%)	1283 (17.7%)	2296 (24.2%)	828 (17.9%)	96,220 (23.5%)
Once or twice a week	102,119 (26.3%)	1449 (20.0%)	2202 (23.2%)	1063 (23.0%)	106,833 (26.1%)
1-3 times a month	43,093 (11.1%)	849 (11.7%)	1103 (11.6%)	522 (11.3%)	45,567 (11.1%)
Special occasions only	42,704 (11.0%)	1026 (14.1%)	1065 (11.2%)	822 (17.8%)	45,617 (11.2%)
Never	27,915 (7.2%)	1632 (22.5%)	1004 (10.6%)	743 (16.1%)	31,294 (7.6%)
Missing	233 (0.1%)	2 (0.0%)	3 (0.0%)	6 (0.1%)	244 (0.1%)
Sedentary time (h/day), mean (SD)	5.1 (2.26)	4.3 (2.23)	4.3 (2.09)	4.5 (2.33)	5.0 (2.26)
Physical activity (MET/min/week), mean (SD)	2912.6 (3220.45)	2850.0 (3040.25)	2896.8 (2938.19)	3288.2 (3329.61)	2915.5 (3212.13)
**Health**					
Multimorbidity, *n* (%)					
No	145,488 (37.5%)	3140 (43.3%)	4217 (44.4%)	1787 (38.6%)	154,632 (37.8%)
Yes	242,243 (62.5%)	4116 (56.7%)	5281 (55.6%)	2838 (61.4%)	254,478 (62.2%)

Multimorbidity was defined as the existence of 2 or more chronic diseases

*BMI* Body mass index, *n* Number, *PA* Physical activity, *MET* Metabolic equivalent, *SD* Standard deviation

The findings for the associations of types of diets with incident cancer are shown in Table 2. In the unadjusted model, vegetarians had a lower risk of liver, pancreatic, lung, prostate, bladder, colorectal, melanoma, kidney, non-Hodgkin lymphoma and lymphatic cancer as well as overall cancer, with hazard ratios ranging from 0.29 to 0.70 (Table 2). However, when the models were fully adjusted for sociodemographic and lifestyle factors, multimorbidity and BMI (model 4), the associations remained statistically significant only for prostate cancer (HR 0.57 [95% CI 0.43 to 0.76]), colorectal cancer (HR 0.73 [95% CI 0.54; 0.99]) and all cancers combined (HR 0.87 [95% CI 0.79 to 0.96]). When colorectal cancer was stratified according to subtypes, a lower risk was observed for colon (HR 0.69 [95% CI 0.48; 0.99]) and proximal colon (HR 0.43 [95% CI 0.22; 0.82]) in vegetarians compared with meat-eaters, but not for rectal or distal cancer.

**Table 2:** Association between the types of diet and cancer incidence for models 0 and 4

Cancer site, model 0 (unadjusted)	Total	Events	Meat-eaters, ref.	Events	Vegan and vegetarian, HR 95% CI	***P*** value	Events	Pescatarian, HR 95% CI	***P*** value	Events	Fish and poultry, HR 95% CI	***P*** value
Overall	409,110	38,042	1.00 (ref.)	463	0.64 (0.58; 0.70)	**< 0.001**	686	0.73 (0.67; 0.78)	**< 0.001**	405	0.89 (0.81; 0.99)	**0.024**
Head and neck	409,110	848	1.00 (ref.)	12	0.76 (0.43; 1.34)	0.341	21	1.01 (0.66; 1.56)	0.960	9	0.89 (0.46; 1.73)	0.740
Oesophagus	409,110	1,010	1.00 (ref.)	11	0.59 (0.32; 1.06)	0.077	7	0.28 (0.13; 0.60)	**0.001**	13	1.09 (0.63; 1.88)	0.764
Stomach	409,110	775	1.00 (ref.)	8	0.55 (0.28; 1.11)	0.096	11	0.58 (0.32; 1.05)	0.072	7	0.76 (0.36; 1.60)	0.472
Colorectal	409,110	4679	1.00 (ref.)	43	0.49 (0.36; 0.66)	**< 0.001**	75	0.65 (0.52; 0.82)	**< 0.001**	42	0.76 (0.56; 1.02)	0.070
Colon	409,110	3340	1.00 (ref.)	29	0.46 (0.32; 0.67)	**< 0.001**	52	0.64 (0.48; 0.84)	**0.001**	29	0.73 (0.51; 1.05)	0.093
Proximal	409,110	1680	1.00 (ref.)	9	0.29 (0.15; 0.55)	**< 0.001**	29	0.70 (0.49; 1.02)	0.061	15	0.75 (0.45; 1.25)	0.272
Distal	409,110	1444	1.00 (ref.)	17	0.63 (0.39; 1.02)	0.059	18	0.51 (0.32; 0.81)	**0.004**	15	0.88 (0.53; 1.46)	0.608
Rectum	409,110	2045	1.00 (ref.)	18	0.47 (0.30; 0.75)	**0.001**	35	0.70 (0.50; 0.98)	**0.035**	24	0.99 (0.66; 1.48)	0.957
Pancreas	409,110	1133	1.00 (ref.)	9	0.43 (0.22; 0.82)	**0.011**	21	0.76 (0.49; 1.17)	0.207	6	0.45 (0.20; 1.00)	**0.049**
Lung	409,110	3306	1.00 (ref.)	27	0.44 (0.30; 0.64)	**< 0.001**	46	0.57 (0.42; 0.76)	**< 0.001**	28	0.71 (0.49; 1.03)	0.075
Melanoma	409,110	1979	1.00 (ref.)	19	0.51 (0.33; 0.81)	**0.004**	28	0.58 (0.40; 0.84)	**0.004**	17	0.72 (0.45; 1.17)	0.184
Breast	218,391	6901	1.00 (ref.)	138	0.85 (0.72; 1.01)	0.065	195	0.85 (0.73; 0.98)	**0.022**	127	1.08 (0.91; 1.29)	0.375
Premenopausal	54,775	1776	1.00 (ref.)	50	1.02 (0.77; 1.36)	0.872	52	0.81 (0.61; 1.07)	0.136	22	0.93 (0.61; 1.41)	0.726
Postmenopausal	130,625	3668	1.00 (ref.)	71	0.86 (0.68; 1.09)	0.211	114	0.89 (0.74; 1.07)	0.210	79	1.05 (0.84; 1.31)	0.678
Uterine	218,391	1132	1.00 (Ref.)	23	0.87 (0.58; 1.31)	0.509	30	0.80 (0.55; 1.14)	0.216	18	0.94 (0.59; 1.49)	0.777
Ovary	218,391	870	1.00 (ref.)	19	0.94 (0.59; 1.47)	0.772	28	0.97 (0.66; 1.41)	0.859	18	1.22 (0.76; 1.94)	0.409
Prostate	190,543	7492	1.00 (ref.)	47	0.46 (0.35; 0.61)	**< 0.001**	82	0.74 (0.59; 0.92)	**0.006**	41	0.88 (0.65; 1.20)	0.422
Kidney	409,110	1227	1.00 (ref.)	12	0.52 (0.30; 0.93)	**0.026**	17	0.57 (0.35; 0.91)	**0.020**	12	0.82 (0.47; 1.46)	0.505
Bladder	409,110	2054	1.00 (ref.)	18	0.47 (0.30; 0.75)	**0.001**	35	0.70 (0.50; 0.97)	**0.033**	16	0.66 (0.40; 1.07)	0.093
Brain	409,110	760	1.00 (ref.)	8	0.56 (0.28; 1.13)	0.107	12	0.64 (0.36; 1.14)	0.131	10	1.11 (0.59; 2.07)	0.745
Haematological	409,110	3,583	1.00 (ref.)	47	0.70 (0.53; 0.94)	**0.016**	66	0.75 (0.59; 0.96)	**0.021**	38	0.89 (0.65; 1.23)	0.490
Non-Hodgkin lymphoma	409,110	1744	1.00 (ref.)	21	0.65 (0.42; 0.99)	**0.046**	27	0.63 (0.43; 0.92)	**0.018**	19	0.92 (0.58; 1.44)	0.712
Multiple myeloma	409,110	921	1.00 (ref.)	13	0.76 (0.44; 1.31)	0.319	21	0.93 (0.60; 1.43)	0.745	11	1.01 (0.56; 1.82)	0.983
Leukaemia	409,110	1116	1.00 (ref.)	16	0.77 (0.47; 1.26)	0.295	25	0.91 (0.62; 1.36)	0.658	11	0.83 (0.46; 1.50)	0.541
**Model 4 (fully adjusted)**												
Overall	409,110	38,042	1.00 (ref.)	463	0.87 (0.79; 0.96)	**0.004**	686	0.93 (0.87; 1.00)	**0.047**	405	0.99 (0.90; 1.09)	0.845
Head and neck	409,110	848	1.00 (ref.)	12	0.94 (0.53; 1.66)	0.824	21	1.23 (0.80; 1.91)	0.344	9	1.05 (0.54; 2.03)	0.889
Oesophagus	409,110	1010	1.00 (ref.)	11	1.13 (0.62; 2.06)	0.679	7	0.51 (0.24; 1.07)	0.075	13	1.64 (0.95; 2.85)	0.076
Stomach	409,110	775	1.00 (ref.)	8	0.94 (0.47; 1.90)	0.870	11	0.99 (0.54; 1.80)	0.967	7	1.08 (0.51; 2.28)	0.836
Colorectal	409,110	4679	1.00 (ref.)	43	0.73 (0.54; 0.99)	**0.042**	75	0.90 (0.71; 1.14)	0.355	42	0.89 (0.66; 1.21)	0.468
Colon	409,110	3340	1.00 (ref.)	29	0.69 (0.48; 0.99)	**0.046**	52	0.87 (0.66; 1.15)	0.321	29	0.84 (0.59; 1.22)	0.367
Proximal	409,110	1680	1.00 (ref.)	9	0.43 (0.22; 0.82)	**0.011**	29	0.96 (0.67; 1.39)	0.847	15	0.84 (0.51; 1.41)	0.515
Distal	409,110	1444	1.00 (ref.)	17	0.94 (0.58; 1.51)	0.784	18	0.70 (0.44; 1.12)	0.141	15	1.07 (0.64; 1.78)	0.805
Rectum	409,110	2045	1.00 (ref.)	18	0.72 (0.45; 1.15)	0.163	35	0.98 (0.70; 1.37)	0.904	24	1.25 (0.83; 1.86)	0.287
Pancreas	409,110	1133	1.00 (ref.)	9	0.65 (0.34; 1.26)	0.204	21	1.08 (0.70; 1.66)	0.741	6	0.52 (0.23; 1.15)	0.108
Lung	409,110	3306	1.00 (ref.)	27	0.76 (0.52; 1.11)	0.150	46	0.86 (0.64; 1.15)	0.300	28	0.81 (0.56; 1.18)	0.282
Melanoma	409,110	1979	1.00 (ref.)	19	0.68 (0.43; 1.07)	0.096	28	0.68 (0.47; 0.98)	**0.041**	17	0.80 (0.50; 1.29)	0.364
Breast	218,391	6895	1.00 (ref.)	138	0.95 (0.80; 1.13)	0.555	194	0.90 (0.78; 1.04)	0.153	127	1.11 (0.93; 1.32)	0.243
Premenopausal	54,775	1776	1.00 (ref.)	50	1.00 (0.75; 1.33)	0.970	52	0.78 (0.59; 1.03)	0.082	22	0.91 (0.60; 1.39)	0.657
Postmenopausal	130,625	3668	1.00 (ref.)	71	0.92 (0.73; 1.16)	0.300	113	0.91 (0.75; 1.10)	0.186	79	1.06 (0.85; 1.33)	0.604
Uterine	218,391	1131	1.00 (ref.)	23	1.15 (0.76; 1.74)	0.522	30	1.09 (0.75; 1.56)	0.655	18	1.07 (0.67; 1.71)	0.764
Ovary	218,391	870	1.00 (ref.)	19	1.14 (0.72; 1.80)	0.583	28	1.07 (0.73; 1.57)	0.716	18	1.20 (0.75; 1.91)	0.455
Prostate	190,543	7492	1.00 (ref.)	47	0.57 (0.43; 0.76)	**< 0.001**	82	0.89 (0.71; 1.11)	0.280	41	0.87 (0.64; 1.18)	0.373
Kidney	409,110	1227	1.00 (ref.)	12	0.88 (0.50; 1.56)	0.662	17	0.93 (0.57; 1.50)	0.765	12	1.14 (0.64; 2.02)	0.652
Bladder	409,110	2054	1.00 (ref.)	18	0.91 (0.57; 1.45)	0.685	35	1.25 (0.89; 1.75)	0.196	16	0.99 (0.61; 1.63)	0.979
Brain	409,110	760	1.00 (ref.)	8	0.73 (0.36; 1.47)	0.379	12	0.78 (0.44; 1.38)	0.394	10	1.25 (0.67; 2.34)	0.485
Thyroid	409,110	287	1.00 (ref.)	8	1.37 (0.68; 2.79)	0.379	6	0.78 (0.35; 1.76)	0.551	3	0.76 (0.24; 2.38)	0.640
Haematological	409,110	3583	1.00 (ref.)	47	0.98 (0.73; 1.31)	0.885	66	1.00 (0.78; 1.28)	0.987	38	1.01 (0.74; 1.40)	0.934
Non-Hodgkin lymphoma	409,110	1744	1.00 (ref.)	21	0.89 (0.58; 1.38)	0.612	27	0.81 (0.55; 1.19)	0.285	19	1.01 (0.64; 1.59)	0.956
Multiple myeloma	409,110	921	1.00 (ref.)	13	0.99 (0.57; 1.72)	0.968	21	1.24 (0.80; 1.92)	0.330	11	1.13 (0.62; 2.04)	0.697
Leukaemia	409,110	1116	1.00 (ref.)	16	1.14 (0.69; 1.87)	0.611	25	1.29 (0.87; 1.93)	0.206	11	0.99 (0.55; 1.80)	0.981

Data presented as adjusted hazard ratio (HR) with 95% confidence interval (95% CI) by type of diet. Meat-eaters were used as the reference group

Model 0 was unadjusted; model 4 included sociodemographic covariates (age, sex, deprivation and ethnicity), lifestyle factors (smoking, alcohol, total physical activity, fruits, and vegetables), comorbidities (presence of 43 diseases), women-reproductive factors, and body mass index; models 1, 2 and 3 are presented in Additional file 1: Table S2

Lower risk of overall cancer and nine cancer sites was found for pescatarians compared with meat-eaters in the unadjusted analyses—kidney, lung, melanoma, non-Hodgkin lymphoma, colorectal (overall and for colon and rectum individually), bladder, prostate, lymphatic and breast—with hazard ratios ranging from 0.57 to 0.65. However, in the maximally adjusted model (model 4), only overall cancer (HR 0.93 [95% CI 0.87 to 1.00]) and melanoma (HR 0.68 [95% CI 0.47; 0.98]) remained significant. The hazard ratios for intermediate models 1, 2 and 3 are presented in Additional file 1: Table S4.

Similar results were found when the models were repeated using the 2-year landmark analysis. In the maximally adjusted models (model 4), the associations for vegetarians were slightly attenuated but remained significant for overall (HR 0.88 [95% CI 0.80 to 0.97]), proximal colon (HR 0.48 [95% CI 0.25; 0.93]) and prostate (HR 0.59 [95% CI 0.44; 0.79]) cancer. However, the associations of vegetarian diet with colorectal and colon cancer were no longer significant. Meanwhile, the associations for pescatarians remained significant for overall cancer (HR 0.93 [95% CI 0.87 to 1.00]) and melanoma (HR 0.68 [95% CI 0.47; 0.98]). There was a lower risk of oesophageal cancer among pescatarians when the 2-year landmark analyses were performed (HR 0.41 [95% CI 0.17; 0.99]) (Additional file 1: Table S5).

### Meta-analysis

**Fig. 1:**
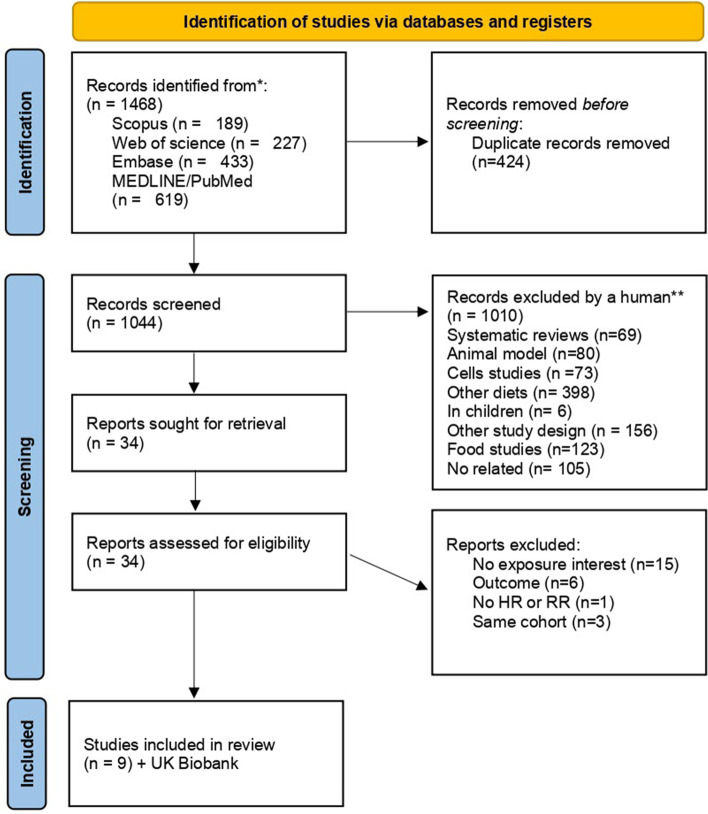
Preferred Reporting Items for Systematic Reviews and Meta-Analyses (PRISMA) flow chart of the study selection process (presented according to the PRISMA guidelines)

A total of 1468 (189 Scopus; 227 Web of Science; 433 EMBASE; 619 PubMed) articles were identified from the search terms. Following the exclusion of duplicates, 1044 abstracts were screened, and 34 manuscripts were reviewed in full. Of these, 25 manuscripts were excluded as they did not meet the inclusion criteria (15 did not report the exposure of interest, 6 did not report cancer outcomes and one did not report relevant effect sizes, i.e. HR or RR). Additionally, three manuscripts were excluded because they reported the same cohort as a later publication. After adding our UK Biobank study, ten studies in total were included in the meta-analysis [13, 15, 17, 31–35] (Fig. 1). Of the 28 cancer sites reported in these papers, only overall cancer and four individual cancer sites could be included in the meta-analysis as they had been reported by at least three independent studies (Additional file 1: Table S6). The definitions used for vegetarian and pescatarian diets in each study are presented in Additional file 1: Table S7. Related to Newcastle-Ottawa bias assessment, most of the studies had moderate risk (Additional file 1: Tables S8 and S9).

**Fig. 2:**
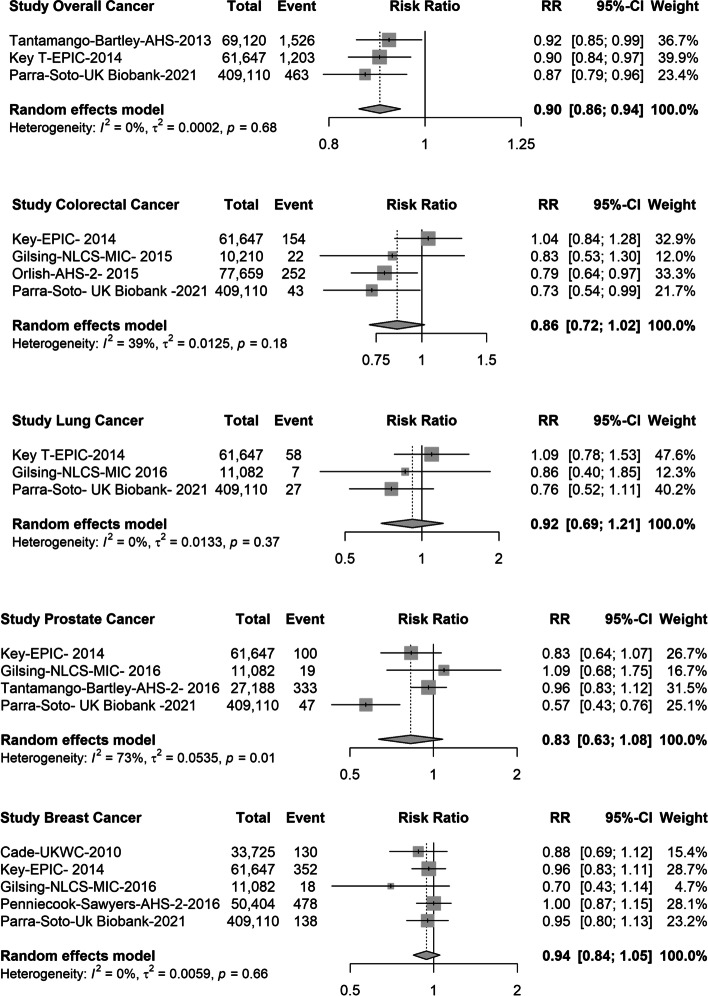
Forest plot of prospective cohort studies evaluating the risk ratios of colorectal, lung, prostate, breast and overall cancer of vegetarians compared with meat-eaters (reference). RR, risk ratio; CI, confidence interval

For vegetarian diets, there were 3 eligible studies (539,877 participants; 3192 events) for overall cancer [13, 15], 4 studies (558,626 participants; 471 events) for colorectal cancer [13, 31, 33], 3 studies (481,839 subjects, 92 events) for lung cancer [13, 32], 4 studies (509,027 participants, 499 events) for prostate cancer [13, 32, 35] and 5 studies (569,968 participants, 1116 events) for breast cancer [13, 17, 32, 34] (Fig. 2). The meta-analysis heterogeneity ranged from 0 to 74% across cancer sites, with the highest heterogeneity found for prostate and colorectal cancer (Fig. 2), and the funnel plots were reasonably symmetrical (Additional file 1: Figs. S2, S3, S4 and S5). For overall cancer, the pooled results suggested a 10% lower risk among vegetarians compared with meat-eaters (RR 0.90, 95% CI 0.86; 0.94), and there was a borderline significant lower risk for colorectal cancer (RR: 0.86, 95% CI: 0.72; 1.02). No associations were observed for lung (RR 0.92, 95% CI 0.69; 1.21), prostate (RR 0.83, 95% CI 0.63; 1.08) or breast cancer (RR: 0.94, 95% CI: 0.84, 1.05) (Fig. 2).

When a sensitivity analysis was performed by replacing vegetarians for lacto-ovo vegetarians defined by Tantamango et al., [15] for overall cancer, Orlish et al [33] for colorectal cancer, and Penniecook et al [34] for breast cancer, similar results were found (Additional file 1: Fig S6). When studies for which the definition of vegetarians included a low meat consumption (less than once a month) were excluded (Orlish et al [33], Tantamango et al., [15] and Penniecook et al [34]) the association of vegetarians with overall cancer remained significant (RR: ;0.89, 95% CI: 0.84; 0.94), while the association for colorectal cancer remained no significant (Additional file 1: Fig S7).

**Fig. 3:**
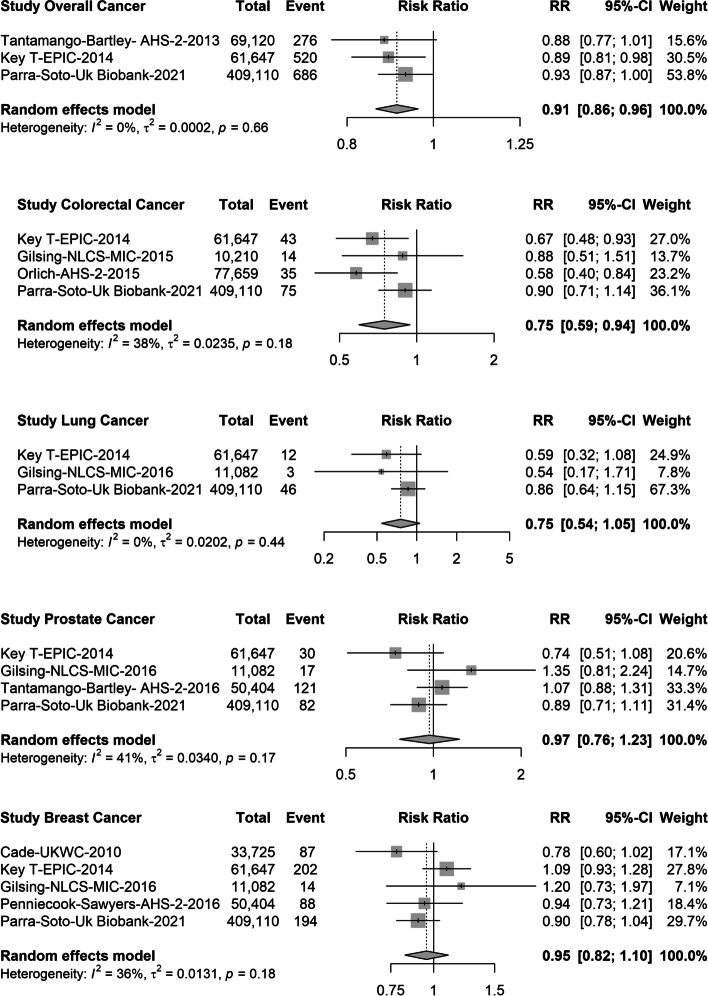
Forest plot of prospective cohort studies evaluating the summary risk ratios of colorectal, lung, prostate, breast and overall cancer of pescatarians compared with meat-eaters (reference). RR, risk ratio; CI, confidence interval

**Fig. 4:**
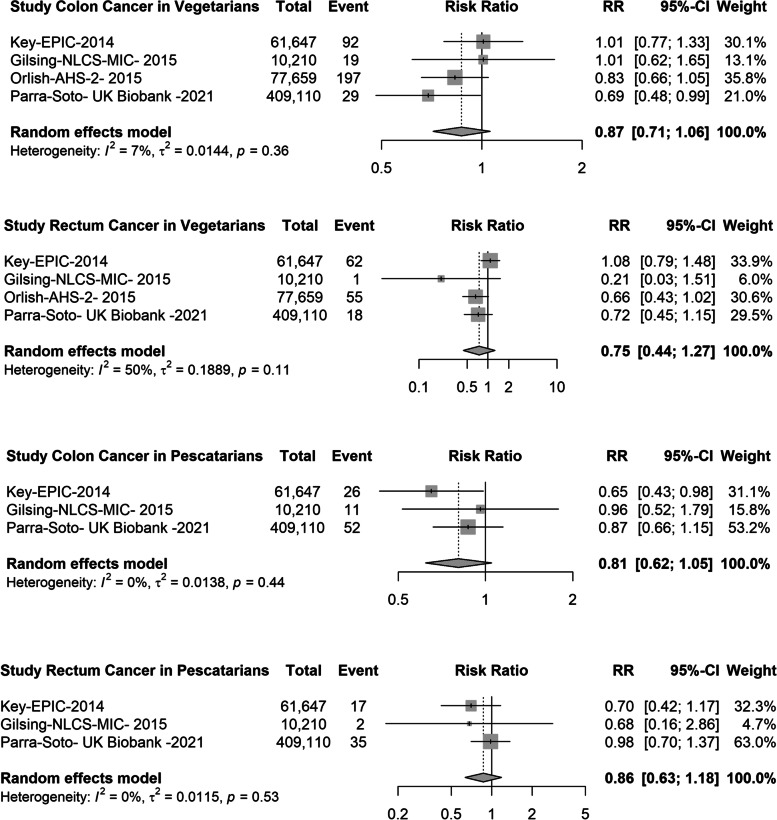
Forest plot of prospective cohort studies evaluating the summary risk ratios of colon and rectal cancer of vegetarians and pescatarians compared with meat-eaters (reference). RR, risk ratio; CI, confidence interval

**Fig. 5:**
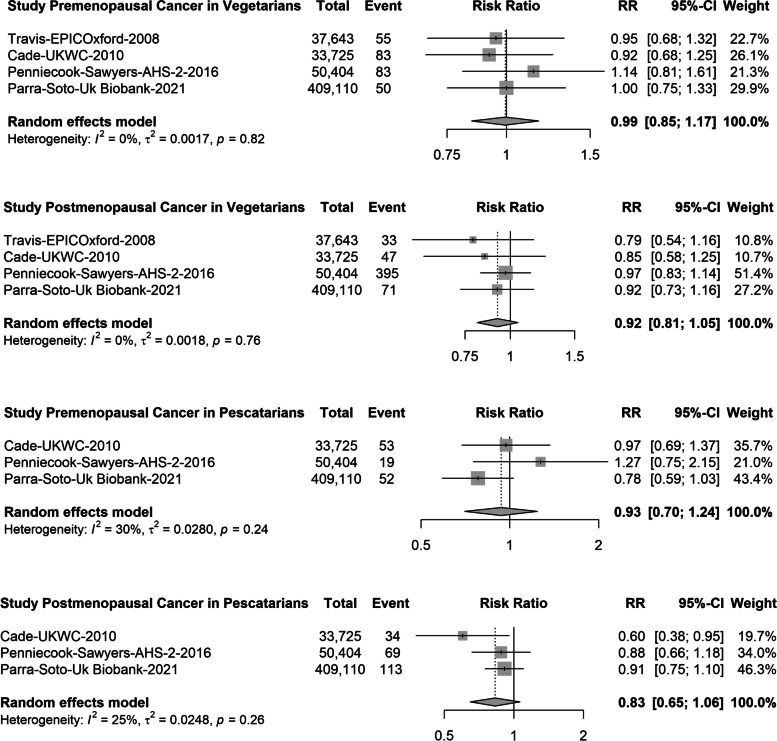
Forest plot of prospective cohort studies evaluating summary risk ratios of breast cancer in premenopausal and postmenopausal women for vegetarians and pescatarians compared with meat-eaters (reference). RR, risk ratio; CI, confidence interval

For pescatarian diets, there were 3 studies (1482 events) for overall cancer [13, 15], 4 studies (167 events) for colorectal cancer [13, 31, 33], 3 studies (61 events) for lung cancer [13, 32], 4 studies (250 events) for prostate cancer [13, 32, 35] and 5 studies (585 events) for breast cancer [13, 17, 32, 34] (Fig. 3). Compared with meat-eaters, pescatarians had a 9% lower risk of overall cancer (pooled RR: 0.91, 95% CI 0.86; 0.96) and a 15% lower risk for colorectal cancer (RR: 0.75, 95% CI: 0.59; 0.94), but no significant associations were observed for lung (RR 0.75, 95% CI 0.54; 1.05), prostate (RR 0.97, 95% CI 0.76; 1.23) or breast cancer (RR: 0.95, 95% CI: 0.82; 1.10) (Fig. 3). When the pooled data were examined for colon and rectal cancer separately, there were no differences in the risk for vegetarians or pescatarians compared with meat-eaters (Fig. 4). Similarly, when breast cancer was stratified by menopausal status, the risk did not differ between vegetarians, pescatarians and meat-eaters (Fig. 5).

Similar results were observed when the study of Penniecook et al [34]) was removed from the the breast cancer analysis on vegetarians, as their definition of vegetarians included a low meat consumption (less than once a month) (Additional file 1: Fig S8) Finally, when Orlish et al [33] was removed from the analysis for colorectal cancer as the authors included low meat consumption (less than once a month) on their definition of pescatarian, the association became non significant (RR: 0.81, 95% CI: 0.65; 1.01) (Additional file 1: Fig S9).

## Discussion

This analysis of the UK Biobank cohort suggests that, compared with﻿ meat-eaters, vegetarians had a lower risk of all cancers combined and of colorectal (especially colon and proximal colon) and prostate cancer and that pescatarians had a lower risk of all cancers and melanoma. These associations were independent of major confounding factors, including socio-demographics, lifestyle, multimorbidity and adiposity. When the results were pooled with eight previous studies in a meta-analysis, the associations with overall and colorectal cancers persisted, but the results relating to other specific cancer sites were inconclusive.

Although evidence for associations between red and processed meat consumption and increased risk of cancer has been widely reported [6, 36–39], there is limited and equivocal evidence for alternative diets, such as vegetarian or pescatarian, on cancer risk [18, 19, 40]. In our study, vegetarians and pescatarians had a 10% and 9% lower risk of overall cancer compared with meat-eaters, respectively. These findings of lower risk agree with evidence reported in previous studies [18]. A meta-analysis published in 2017, which included 38,033 participants from two prospective cohort studies from the USA and the UK, of whom 1976 developed incident all-cause cancers, showed that vegetarians had an 8% lower risk [18]. However, an earlier meta-analysis by Huang et al. in 2012, which included 124,706 participants from the UK, the Netherlands and Germany, reported a larger reduction of 18% for overall cancer incidence in vegetarians compared with meat-eaters [41].

Regarding specific cancers, in a study including 77,659 participants who were followed up for 7.3 years, Orlich et al. reported that vegetarians had a 22% lower incidence risk of colorectal cancer (HR 0.78 [95% CI, 0.64; 0.95] (490 cases)) compared with meat-eaters. The reduction in risk was stronger for pescatarians, who had a 43% lower risk of colorectal cancer (HR 0.57 [95% CI, 0.40; 0.82]) [33]. In a relatively recent meta-analysis, Godos et al. [19], which included a total of nine studies including six cohorts accounting for 686,629 individuals, and 3441, 4062 and 1935 cases of breast, colorectal and prostate cancer, respectively, reported no significant differences between vegetarians and non-vegetarians in the risk of breast and prostate cancer. However, they did find a lower risk of colorectal cancer in semi-vegetarians (RR 0.86 [95% CI 0.79; 0.94]) and pescatarians (RR 0.67 [95% CI 0.53; 0.83]) [19]. In our study using UK Biobank data, we found a 27% lower risk of colorectal cancer in vegetarians. When the analyses were stratified by subtype, cancers in the proximal colon and colon showed a 57% and 31% lower risk in vegetarians, respectively, with no associations observed for distal colon or rectal cancer. Interestingly, for pescatarians, we observed significant associations for colorectal and colon cancer only in the minimally adjusted model, and these associations were completely attenuated when the analyses were adjusted for socio-demographics, lifestyle and BMI. Similar to breast and lung cancers, the associations with diet type were significant in the unadjusted model but disappeared completely in the most adjusted model. Indeed, these results are in agreement with previous studies, especially for breast cancer, where associations were attenuated after accounting for BMI [13, 17, 32, 34]. No studies have reported significant differences in lung cancer risk for vegetarians or pescatarians versus meat-eaters in maximally adjusted models [13, 17, 32]. Our finding of no significant associations with site-specific cancers among poultry eaters was similar to those reported by Cade et al. in 2010 [17].

Some of the mechanisms that could explain the associations between vegetarian diet and cancer risk are the presence of bioactive compounds in plant-based diets, such as fibre, phenol, polyphenol, and sulphuric compounds, and other antioxidants compounds, including vitamins. These compounds have been shown to have anti-carcinogenic effects in experimental models and epidemiological studies [42, 43]. On the other hand, this dietary pattern has some deficient intake of certain nutrients such as iron and vitamin B12. For instance, long-chain n-3 fatty acids, such as eicosapentaenoic acid and docosahexaenoic acid, are lower in vegetarians [44]. Therefore, decreased intakes of some of these nutrients have been related to a higher cancer incidence in some studies [45]. We cannot discard that some of the associations described may be mediated through other risk factors such as adiposity [46–48] or smoking [48].

It is important to notice that not all vegetarians’ diets are healthy [49]; higher consumption of ultra-processed food could reduce the benefit of a vegetarian diet on cancer risk. In our sample, vegetarian and pescatarians reported eating more crisps and pizza than meat-eaters.

Despite low/moderate heterogeneity in our meta-analysis, studies differed in a variety of ways that could lead to inconsistencies in their findings. Some of these include the length of follow-up (ranging from 4.1 to 20.3 years), differences in confounding factors controlled for in each study, differences in sample size across cohorts (ranging from 10,210 to 409,110) and risks of bias attributable to the design of the studies which varied from low to moderate [18]. Studies also differed in how vegetarian and other types of diets were defined and for how long participants had been following their attributed type of diet [41]. However, the measured heterogeneity between studies included in the meta-analyses was low.

### Strength and limitations

UK Biobank is a large, prospective, general population cohort with data on diet and a wide range of potential confounders and health outcomes. However, the UK Biobank is not representative of the general UK population regarding lifestyle and baseline health [50]. Moreover, the participants who have full data available and therefore were included in this study were leaner and more affluent than those excluded due to missing data and prevalent cancer at baseline (Additional file 1: Table S3). Regardless of these differences, our risk estimates were similar to other more population-representative cohorts [20]. In the UK Biobank, dietary data were collected from all participants on one occasion; therefore, we cannot rule out that the type of diet reported was not modified over the length of the study. However, an analysis of the repeatability of the touchscreen questionnaire in a sub-set of participants (*n* = 20.348), who repeated the assessment centre visit approximately 4 years after recruitment, showed that the dietary touchscreen variables, available for the full cohort, reliably rank participants according to intakes of the main food groups. In our study, we were unable to investigate the association of vegan diet with cancer risk because there were very small numbers (*n* = 57) of participants who were vegan. Also, we were not able to include energy intake as a covariate as data were available for only half of the cohort population. Moreover, exposure specificity is also a limitation as there was a lack of data on what alternative nutrients or food sources were used to replace meat or fish consumption. An additional limitation of the UK Biobank findings is that data were collected at a single time point resulting in the inability to properly adjust for changes in the exposure or covariates over time.

For the meta-analysis, some data limitations should be considered when interpreting the results. The only available evidence was from high-income countries in Europe and North America, i.e. the USA, Canada, the UK and the Netherlands. Therefore, associations between dietary patterns and cancer in other continents and in low-income and middle-income countries remain to be investigated. The number of available studies was generally low for the cancer-specific analyses. This limited the possibility of exploring subgroup analysis, but the heterogeneity across studies was relativity low. The definitions used for different types of diets differed between studies, which could introduce bias and reduce the likelihood of detecting associations. Future research in this area should aim to standardize diet classifications relating to the types of food consumed and their frequency of consumption.

## Conclusion

Our UK Biobank findings suggest that, compared with meat-eaters, vegetarians had a lower risk of cancer overall, probably due to a lower risk of colorectal and prostate cancer. Pescatarians also had a lower overall risk of cancer, but the relationships with specific contributory cancers were unclear. However, when our risk estimates were pooled with those from previous prospective cohort studies, there was no conclusive evidence in relation to site-specific cancers, even though the associations with overall cancer risk were significant. Larger studies with longer follow-up periods and better classification of diet types are needed to elucidate the benefits, or otherwise, of vegetarian and pescatarian diets on the risk of individual cancers.

## Supplementary Information

**Additional file 1: Methods**. **Fig. S1.** Flowchart of UK Biobank participants. **Table S1.** Criteria for the Newcastle-Ottawa Scale regarding star allocation to assess quality of studies. **Table S2.** Details of dietary characteristics for 175,499 UK Biobank participants with data available for the 24 hrs diet recall. **Table S3.** Baseline characteristics people with missing data who were not included in the study. **Table S4.** Association between types of diet and cancer incidence for Models 1-3. **Table S5.** Landmark sensitivity analysis: Association between types of diet and cancer incidence for all Models after excluding events in the first 2 years of follow-up. **Table S6.** Characteristics of the cohorts included in systematic review. **Table S7.** Definitions of vegetarian, lacto-ovo-vegetarian, pescatarian and poultry diets used in the various studies. **Table S8.** Quality assessment of studies using a modified Newcastle-Ottawa scale for assessing studies in the systematic review of vegetarian diet and cancer risk. **Table S9.** Risk of bias assessment. **Fig. S2.** Funnel plot of prospective cohort studies evaluating summary hazard ratios of colorectal, lung, prostate, breast and overall cancer for vegetarians versus meat-eaters. **Fig. S3.** Funnel plot of prospective cohort studies evaluating summary hazard ratios of colorectal, lung, prostate, breast and overall cancer for pescatarians versus meat-eaters. **Fig. S4.** Funnel plot of prospective cohort studies evaluating summary hazard ratios of colon and rectum cancer for vegetarian and pescatarians versus meat-eaters. **Fig. S5.** Funnel plot of prospective cohort studies evaluating summary hazard ratios of breast cancer in premenopausal and postmenopausal for vegetarian and pescatarians versus meat-eaters. **Fig S6.** Sensitivity analysis of prospective cohort studies evaluating summary risk ratios of of Lacto-Ovo vegetarians defined by Tantamango et al. for overall cancer, Orlish et al. for colorectal cancer, and Penniecook et al for breast cancer, compared with non-vegetarians (reference). **Fig S7.** Sensitivity analysis of prospective cohort studies evaluating summary risk ratios of overall, colorectal, lung, prostate and breast cancer for vegetarians compared with meat-eaters. **Fig S8.** Sensitivity analysis of prospective cohort studies evaluating summary risk ratios of breast cancer in premenopausal and postmenopausal women for vegetarians compared with meat-eaters (reference). **Fig S9.** Sensitivity analysis of prospective cohort studies evaluating summary risk ratios of colorectal, lung, prostate, breast and overall cancer of pescatarians compared with meat-eaters.

## Data Availability

This research has been conducted using the UK Biobank Resource under Application Number 7155. The UK Biobank is an open-access resource, and all bona fide researchers can use it for approved research by registering and applying at http://www.ukbiobank.ac.uk/register-apply/.

## Abbreviations

BMI: Body mass index
CI: Confidence interval
HR: Hazard ratio
MET: Metabolic equivalent
*n*: Number
PA: Physical activity
RR: Relative risk
SD: Standard deviation
