# Supplementary material for: Association of meat, vegetarian, pescatarian and fish-poultry diets with risk of 19 cancer sites and all cancer: findings from the UK Biobank prospective cohort study and meta-analysis
Source: BMC Med. 2022 Feb 24;20:79. doi: 10.1186/s12916-022-02257-9 (PMC9281076; doi:10.1186/s12916-022-02257-9)
Supplement: Supplementary file 1 — Additional file 1: Methods. Fig. S1. Flowchart of UK Biobank participants. Table S1. Criteria for the Newcastle-Ottawa Scale regarding star allocation to assess quality of studies. Table S2. Details of dietary characteristics for 175,499 UK Biobank participants with data available for the 24 hrs diet recall. Table S3. Baseline characteristics people with missing data who were not included in the study. Table S4. Association between types of diet and cancer incidence for Models 1-3. Table S5. Landmark sensitivity analysis: Association between types of diet and cancer incidence for all Models after excluding events in the first 2 years of follow-up. Table S6. Characteristics of the cohorts included in systematic review. Table S7. Definitions of vegetarian, lacto-ovo-vegetarian, pescatarian and poultry diets used in the various studies. Table S8. Quality assessment of studies using a modified Newcastle-Ottawa scale for assessing studies in the systematic review of vegetarian diet and cancer risk. Table S9. Risk of bias assessment. Fig. S2. Funnel plot of prospective cohort studies evaluating summary hazard ratios of colorectal, lung, prostate, breast and overall cancer for vegetarians versus meat-eaters. Fig. S3. Funnel plot of prospective cohort studies evaluating summary hazard ratios of colorectal, lung, prostate, breast and overall cancer for pescatarians versus meat-eaters. Fig. S4. Funnel plot of prospective cohort studies evaluating summary hazard ratios of colon and rectum cancer for vegetarian and pescatarians versus meat-eaters. Fig. S5. Funnel plot of prospective cohort studies evaluating summary hazard ratios of breast cancer in premenopausal and postmenopausal for vegetarian and pescatarians versus meat-eaters. Fig S6. Sensitivity analysis of prospective cohort studies evaluating summary risk ratios of of Lacto-Ovo vegetarians defined by Tantamango et al. for overall cancer, Orlish et al. for colorectal cancer, and Penniecook et al for breast cancer, compa [file 12916_2022_2257_MOESM1_ESM.docx]

**Additional file 1: Parra-Soto S., Association of meat, vegetarian, pescatarian, and fish-poultry eaters with risk of 19 cancer sites: Findings from the UK Biobank prospective cohort study and meta-analysis.**

**Methods**

**Key search terms used in the systematic review**

#1 Vegetarian, #2 Vegetarianism, #3 "Vegetarian Diet", #4Vegan, #5Veganism, #6 "Vegan Diet", #7Veg$, #8 "Plant-Based", #9 Cancer, #10Neoplasms, #11 Cohort, #12 cohort Study, #13Prospective, #14 "Prospective Cohort Study", #15 Incidence, #16 "Incidence Studies" and MESH terms: #17 "Diet, vegetarian", #18 "Neoplasm" y #19 "cohort studies. They were combined in the following the following search codes: ((#1 OR #2 OR #3 OR #4 OR #5 OR #6 OR #7 OR #8 OR #17) AND (#9 OR #10 OR #18) AND (#11 OR #12 OR #13 OR #14 OR #15 OR #16 OR #19)).

**EMBASE**

(vegetarian:ab,ti OR vegetarianism OR vegan OR veganism OR (vegan AND diet) OR (plant AND based) OR (vegetarian AND diet)) AND (cancer OR 'neoplasm') AND ('cohort analysis' OR (cohort AND study) OR (retrospective AND cohort AND study) OR (retrospective AND cohort) OR (retrospective AND cohort AND study) OR (prospective AND cohort AND study) OR (prospective AND cohort) OR (incidence AND studies)) AND ([article]/lim OR [article in press]/lim) AND ([adult]/lim OR [middle aged]/lim OR [aged]/lim OR [very elderly]/lim) AND [humans]/lim AND [embase]/lim

**WEB OF SCIENCE**

AB = (vegetarian OR vegetarianism OR vegan OR veganism OR "vegan diet" OR "plant based"OR "vegetarian diet)" AND cancer OR neoplasm AND "cohort analysis" OR "cohort study" OR "retrospective cohort study" OR "retrospective cohort" OR "retrospective cohort study" OR "prospective cohort study" OR "prospective cohort" OR "incidence studies")

AND AB = ( cancer OR neoplasm) AND AB = ("cohort" OR "cohort study")

**SCOPUS**

TITLE-ABS-KEY ( ( vegetarian OR vegetarianism OR vegan OR veganism OR "plant based" OR "vegetarian diet" AND cancer OR neoplasm AND "cohort" OR "cohort study" OR retrospective ) ) AND ( LIMIT-TO ( DOCTYPE , "ar" ) )


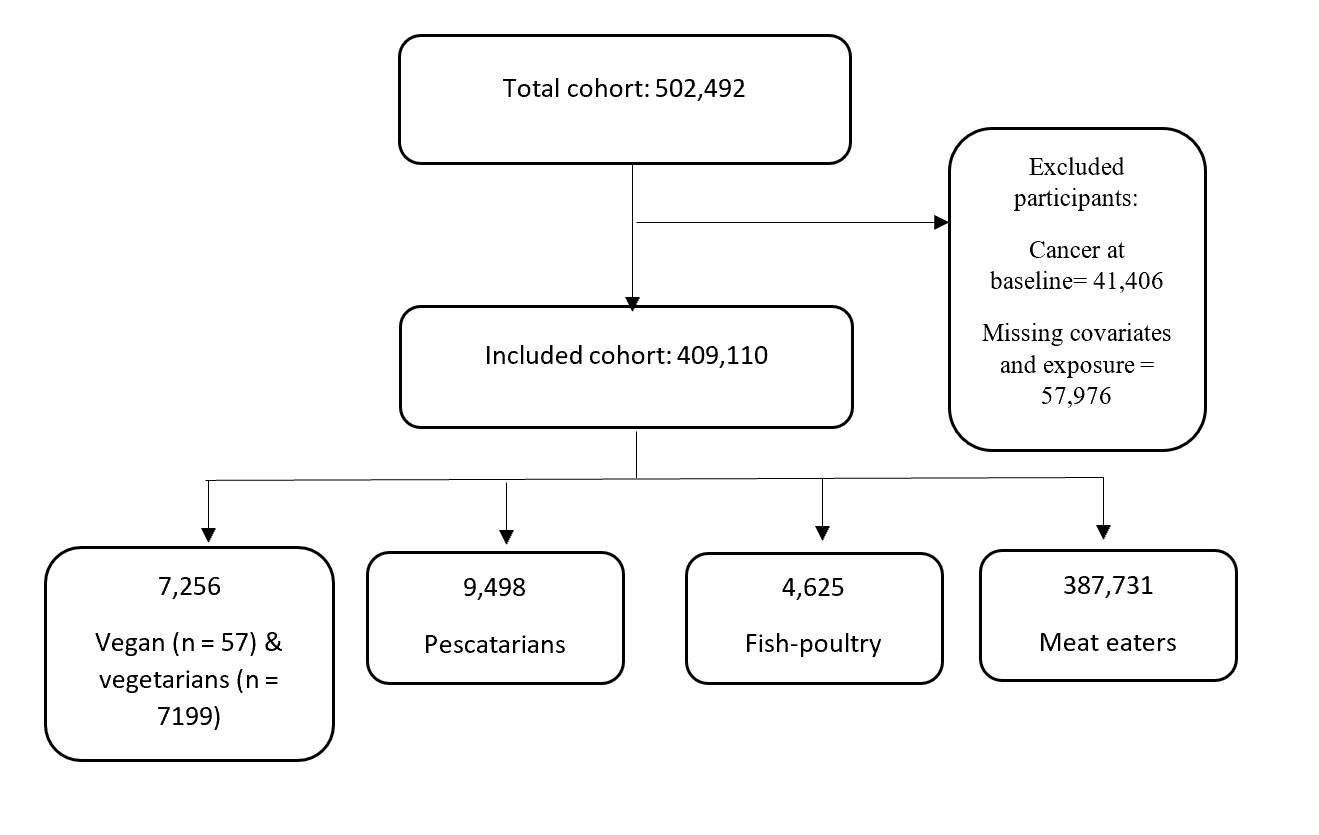


Fig. S1: Flowchart of UK Biobank participants.

Table S1: Criteria for the Newcastle-Ottawa Scale regarding star allocation to assess quality of studies (out of a total of seven stars).

| \| **Criteria** \| \| --- \| | \| **Acceptable (star awarded):** \| \| --- \| | \| **Unacceptable (star not awarded):** \| \| --- \| |
| --- | --- | --- | --- | --- | --- |
| \| *Representativeness of exposed cohort* \| \| --- \| | \| Population-based \| \| --- \| | \| Hospital-based \| \| --- \| |
| \| *Selection of non-exposed cohort* \| \| --- \| | \| Same setting as exposed cohort \| \| --- \| | \| Different setting from exposed cohort \| \| --- \| |
| \| *Ascertainment of exposure* \| \| --- \| | \| Secure records or directly measured \| \| --- \| | \| Self-reported information \| \| --- \| |
| \| *Comparability* \| \| --- \| | \| Excluded or adjusted for prior outcome in analysis \| \| --- \| | \| No exclusion of prior outcome \| \| --- \| |
|  | \| Adjusted for age, race, smoking \| \| --- \| | \| Did not adjust for age, race, smoking \| \| --- \| |
| \| *Outcome of interest* \| \| --- \| | Secure records or directly measured | \| Self-reported information \| \| --- \| |
| \| *Adequacy of follow-up* \| \| --- \| | \| Adjusted for missing data or follow-up > 1 month. \| \| --- \|  \|  \| \| --- \| | \| No statement regarding missing data. No follow-up after birth \| \| --- \| |

Table S2: Details of dietary characteristics for 175,499 UK Biobank participants.

|  | **Meat-eaters** | **Vegan & vegetarian** | **Pescatarian** | **Fish & poultry** | **Overall** |
| --- | --- | --- | --- | --- | --- |
| N (%) | 164,492 (93.7%) | 3,846 (2.2%) | 5,159 (2.9%) | 2,002 (1.1%) | 175,499 |
| Total energy intake (kcal/day), mean (SD) | 2123.7 (645.76) | 2071.2 (717.53) | 2074.7 (655.92) | 1985.1 (699.11) | 2119.5 (648.60) |
| CHO intake (% of TE), mean (SD) | 47.0 (8.04) | 52.1 (8.11) | 50.1 (8.06) | 50.3 (8.53) | 47.3 (8.10) |
| Sugar intake (% of TE), mean (SD) | 22.4 (6.94) | 24.1 (7.34) | 23.7 (7.02) | 25.1 (7.63) | 22.5 (6.97) |
| Fibre intake (g/day), mean (SD) | 16.4 (7.62) | 21.0 (9.22) | 20.0 (8.90) | 19.5 (9.63) | 16.6 (7.78) |
| Protein intake (% of TE), mean (SD) | 15.7 (3.61) | 12.4 (2.31) | 13.5 (2.71) | 15.2 (3.52) | 15.5 (3.61) |
| Fat intake (% of TE), mean (SD) | 32.1 (6.68) | 31.7 (7.15) | 31.9 (6.97) | 30.8 (7.27) | 32.0 (6.71) |
| Polyunsaturated fat intake (% of TE), mean (SD) | 5.9 (2.22) | 6.3 (2.47) | 6.3 (2.34) | 6.0 (2.27) | 5.9 (2.23) |
| Saturated fat intake (% of TE), mean (SD) | 12.4 (3.31) | 12.0 (3.60) | 11.8 (3.40) | 11.3 (3.53) | 12.3 (3.32) |
| Vitamin D, (ug), mean (SD)* | 2.8 (3.29) | 1.2 (1.90) | 3.3 (4.34) | 3.14 (4.22) | 2.8 (3.32) |
| Iron, (mg), mean (SD)* | 13.6 (5.23) | 14.3 (6.15) | 14.6 (5.74) | 13.74 (5.77) | 13.7 (5.28) |
| Fruit and vegetable intake (g/day), mean (SD) | 328.3 (181.81) | 403.8 (223.13) | 406.7 (212.51) | 426.0 (229.14) | 333.4 (185.44) |
| Water intake (glasses/day), mean (SD) | 2.9 (2.20) | 3.4 (2.66) | 3.4 (2.43) | 3.6 (2.57) | 2.9 (2.22) |
| **Fried potatoes** |  |  |  |  |  |
| < quarter portion | 121,425 (74.6%) | 3,114 (81.4%) | 4,181 (81.8%) | 1,676 (84.4%) | 130,396 (75.1%) |
| Between quarter portion and Half portion | 7,130 (4.4%) | 133 (3.5%) | 171 (3.3%) | 67 (3.4%) | 7,501 (4.3%) |
| Between Half portion and 1 portion | 30,680 (18.9%) | 509 (13.3%) | 686 (13.4%) | 219 (11.0%) | 32,094 (18.5%) |
| Between 1 portion and 3 portions | 2,636 (1.6%) | 56 (1.5%) | 55 (1.1%) | 19 (1.0%) | 2,766 (1.6%) |
| More than 2 portions | 803 (0.5%) | 14 (0.4%) | 18 (0.4%) | 5 (0.3%) | 840 (0.5%) |
| **Fizzy drinks** |  |  |  |  |  |
| < 1 portion | 157,865 (97.0%) | 3,734 (97.6%) | 5,021 (98.2%) | 1,949 (98.1%) | 168,569 (97.1%) |
| Between 1 portion and 2 portions | 3,642 (2.2%) | 74 (1.9%) | 71 (1.4%) | 27 (1.4%) | 3,814 (2.2%) |
| Between 2 portion and 3 portions | 782 (0.5%) | 9 (0.2%) | 9 (0.2%) | 7 (0.4%) | 807 (0.5%) |
| More than 3 portions | 385 (0.2%) | 9 (0.2%) | 10 (0.2%) | 3 (0.2%) | 407 (0.2%) |
| **Crisp** |  |  |  |  |  |
| < half portion | 130,692 (80.3%) | 2,965 (77.5%) | 4,172 (81.6%) | 1,711 (86.2%) | 139,540 (80.4%) |
| Between half portion and 1 portion | 27,813 (17.1%) | 739 (19.3%) | 800 (15.7%) | 239 (12.0%) | 29,591 (17.0%) |
| Between 1 portion and 3 portions | 3,802 (2.3%) | 108 (2.8%) | 124 (2.4%) | 30 (1.5%) | 4,064 (2.3%) |
| More than 3 portions | 367 (0.2%) | 14 (0.4%) | 15 (0.3%) | 6 (0.3%) | 402 (0.2%) |
| **Pizza** |  |  |  |  |  |
| < quarter portion | 155,165 (95.4%) | 3,530 (92.3%) | 4,803 (94.0%) | 1,903 (95.8%) | 165,401 (95.3%) |
| Between quarter portion and Half portion | 1,375 (0.8%) | 39 (1.0%) | 46 (0.9%) | 7 (0.4%) | 1,467 (0.8%) |
| Between Half portion and 3 portion | 2,206 (1.4%) | 84 (2.2%) | 91 (1.8%) | 28 (1.4%) | 2,409 (1.4%) |
| More than 3 portions | 2,376 (1.5%) | 101 (2.6%) | 111 (2.2%) | 30 (1.5%) | 2,618 (1.5%) |
| Missing | 1553 (1.0%) | 72 (1.9%) | 60 (1.2%) | 18 (0.9%) | 1703 (1.0%) |

*Available for 58,496

Table S3: Baseline characteristics people with missing data who were not included in the study.

|  | **No included (N=57,976)** | **Included**  **(N=409,110)** |
| --- | --- | --- |
| **Age, mean (SD)** | 56.2 (8.17) | 56.3 (8.11) |
| **Sex, n (%)** |  |  |
| Females | 32,192 (55.5%) | 218,567 (53.4%) |
| Males | 25,768 (44.4%) | 190,543 (46.6%) |
| **Townsend deprivation index, n(%)** |  |  |
| Lower deprivation | 16,143 (27.7 %) | 139,244 (34.0%) |
| Middle | 17,798 (30.5 %) | 137,590 (33.6%) |
| Higher deprivation | 23,692 (40.7 %) | 132,276 (32.3%) |
| Missing | 639 (1.1 %) | - |
| **Ethnicity, n (%)** |  |  |
| White | 51,289 (88.0 %) | 387,366 (94.7%) |
| Mixed | 1,527 (2.6 %) | 5,663 (1.4%) |
| South Asian | 2,059 (3.5 %) | 7,512 (1.8%) |
| Black | 1,691 (2.9 %) | 6,079 (1.5%) |
| Chinese | 282 (0.5 %) | 1,230 (0.3%) |
| Missing | 1,424 (2.4 %) | 1260 (0.3%) |
| **Nutritional status** |  |  |
| Height (m), mean (SD) | 1.7 (0.09) | 1.68 (0.09) |
| Weight (Kg), mean (SD) | 79.9 (17.01) | 78.0 (15.82) |
| Waist (cm), mean (SD) | 92.3 (14.10) | 90.1 (13.38) |
| Body Mass index (kg/m^2^), mean (SD) | 28.3 (5.26) | 27.3 (4.72) |
| BMI classification**, n (%)** |  |  |
| Underweight | 304 (0.5 %) | 2,096 (0.5%) |
| Normal | 15,331 (26.3 %) | 135,399 (33.1%) |
| Overweight | 22,730 (39.0 %) | 174,881 (42.7%) |
| Obese | 16,972 (29.1 %) | 96,734 (23.6%) |
| Missing | 2,935 (5.0 %) | - |
| **Smoking, n (%)** |  |  |
| Never | 28,483 (48.9 %) | 227,139 (55.5%) |
| Previous | 18,805 (32.3 %) | 140,398 (34.3%) |
| Current | 8,035 (13.8 %) | 41,573 (10.2%) |
| Missing | 2,949 (5.1 %) | - |
| **Alcohol intake, n (%)** |  |  |
| Daily or almost daily | 11,160 (19.2 %) | 83,335 (20.4%) |
| 3-4 times a week | 11,653 (20.0 %) | 96,220 (23.5%) |
| Once or twice a week | 13,659 (23.4 %) | 106,833 (26.1%) |
| 1-3 times a month | 6,452 (11.1 %) | 45,567 (11.1%) |
| Special occasions only | 7,819 (13.4 %) | 45,617 (11.2%) |
| Never | 6,278 (10.8 %) | 31,294 (7.6%) |
| Missing | 1,251 (2.1 %) | 244 (0.1%) |
| **Sedentary time (h/day)**, mean (SD) | 5.0 (2.54) | 5.0 (2.26) |
| **Physical activity (MET/min/week)** | 3138.8 (3534.36) | 2915.5 (3212.13) |
| **Multimorbidity, n (%)** |  |  |
| No | 18,467 (32.7 %) | 154,632 (37.8%) |
| Yes | 39,804 (68.3 %) | 254,478 (62.2%) |

Table S4: Association between types of diet and cancer incidence for Models 1-3.

| **Cancer site** | **Total** | **Events** | **Meat-eaters** | **Events** | **Vegan & Vegetarian** |  | **Events** | **Pescatarian** |  | **Events** | **Fish & Poultry** |  |
| --- | --- | --- | --- | --- | --- | --- | --- | --- | --- | --- | --- | --- |
| **Model 1** |  |  |  |  | **HR 95% CI** | P value |  | **HR 95% CI** | P value |  | **HR 95% CI** | P value |
| Overall | 409,110 | 38,042 | 1.00 (Ref.) | 463 | 0.83 (0.76; 0.91) | **<0.001** | 686 | 0.89 (0.82; 0.95) | **0.002** | 405 | 0.95 (0.86; 1.05) | 0.310 |
| Head & neck | 409,110 | 848 | 1.00 (Ref.) | 12 | 0.91 (0.51; 1.61) | 0.740 | 21 | 1.22 (0.79; 1.88) | 0.379 | 9 | 1.03 (0.54; 2.00) | 0.919 |
| Oesophagus | 409,110 | 1,010 | 1.00 (Ref.) | 11 | 0.97 (0.53; 1.76) | 0.919 | 7 | 0.43 (0.20; 0.90) | **0.025** | 13 | 1.43 (0.82; 2.47) | 0.203 |
| Stomach | 409,110 | 775 | 1.00 (Ref.) | 8 | 0.83 (0.41; 1.66) | 0.594 | 11 | 0.85 (0.47; 1.55) | 0.605 | 7 | 0.96 (0.45; 2.02) | 0.908 |
| Colorectal | 409,110 | 4,679 | 1.00 (Ref.) | 43 | 0.70 (0.51; 0.94) | **0.018** | 75 | 0.85 (0.68; 1.07) | 0.171 | 42 | 0.85 (0.63; 1.16) | 0.310 |
| Colon | 409,110 | 3,340 | 1.00 (Ref.) | 29 | 0.65 (0.45; 0.94) | **0.021** | 52 | 0.82 (0.62; 1.08) | 0.152 | 29 | 0.80 (0.56; 1.16) | 0.241 |
| Proximal | 409,110 | 1,680 | 1.00 (Ref.) | 9 | 0.40 (0.21; 0.77) | **0.006** | 29 | 0.89 (0.62; 1.29) | 0.548 | 15 | 0.79 (0.48; 1.32) | 0.375 |
| Distal | 409,110 | 1,444 | 1.00 (Ref.) | 17 | 0.89 (0.55; 1.43) | 0.619 | 18 | 0.67 (0.42; 1.07) | 0.091 | 15 | 1.02 (0.61; 1.69) | 0.949 |
| Rectum | 409,110 | 2,045 | 1.00 (Ref.) | 17 | 0.68 (0.43; 1.09) | 0.110 | 35 | 0.94 (0.67; 1.31) | 0.712 | 24 | 1.19 (0.80; 1.78) | 0.392 |
| Pancreas | 409,110 | 1,133 | 1.00 (Ref.) | 9 | 0.60 (0.31; 1.16) | 0.129 | 21 | 0.99 (0.64; 1.52) | 0.959 | 6 | 0.48 (0.22; 1.07) | 0.074 |
| Lung | 409,110 | 3,306 | 1.00 (Ref.) | 27 | 0.62 (0.43; 0.91) | **0.014** | 46 | 0.72 (0.54; 0.96) | **0.025** | 28 | 0.70 (0.48; 1.01) | 0.060 |
| Melanoma | 409,110 | 1,979 | 1.00 (Ref.) | 19 | 0.68 (0.43; 1.07) | 0.095 | 28 | 0.67 (0.46; 0.98) | **0.039** | 17 | 0.80 (0.50; 1.29) | 0.358 |
| Breast | 218,391 | 6,901 | 1.00 (Ref.) | 138 | 0.93 (0.79; 1.10) | 0.400 | 195 | 0.89 (0.77; 1.02) | 0.104 | 127 | 1.09 (0.92; 1.30) | 0.328 |
| Premenopausal | 54,775 | 1,776 | 1.00 (Ref.) | 50 | 1.02 (0.77; 1.36) | 0.872 | 52 | 0.81 (0.61; 1.07) | 0.136 | 22 | 0.93 (0.61; 1.41) | 0.726 |
| Postmenopausal | 130,625 | 3,668 | 1.00 (Ref.) | 71 | 0.86 (0.68; 1.09) | 0.211 | 114 | 0.89 (0.74; 1.07) | 0.210 | 79 | 1.05 (0.84; 1.31) | 0.678 |
| Uterine | 218,391 | 1,132 | 1.00 (Ref.) | 23 | 1.03 (0.68; 1.55) | 0.907 | 30 | 0.90 (0.62; 1.29) | 0.550 | 18 | 0.93 (0.58; 1.48) | 0.757 |
| Ovary | 218,391 | 870 | 1.00 (Ref.) | 19 | 1.17 (0.74; 1.84) | 0.507 | 28 | 1.11 (0.76; 1.61) | 0.597 | 18 | 1.23 (0.77; 1.96) | 0.388 |
| Prostate | 190,543 | 7,492 | 1.00 (Ref.) | 47 | 0.59 (0.44; 0.78) | **<0.001** | 82 | 0.92 (0.74; 1.14) | 0.446 | 41 | 0.90 (0.66; 1.22) | 0.484 |
| Kidney | 409,110 | 1,227 | 1.00 (Ref.) | 12 | 0.76 (0.43; 1.35) | 0.358 | 17 | 0.79 (0.49; 1.28) | 0.343 | 12 | 1.01 (0.57; 1.78) | 0.976 |
| Bladder | 409,110 | 2,054 | 1.00 (Ref.) | 18 | 0.82 (0.51; 1.30) | 0.394 | 35 | 1.13 (0.81; 1.58) | 0.484 | 16 | 0.90 (0.55; 1.48) | 0.691 |
| Brain | 409,110 | 760 | 1.00 (Ref.) | 8 | 0.74 (0.37; 1.48) | 0.389 | 12 | 0.79 (0.45; 1.40) | 0.416 | 10 | 1.26 (0.67; 2.35) | 0.473 |
| Haematological | 409,110 | 3,583 | 1.00 (Ref.) | 47 | 0.95 (0.71; 1.27) | 0.731 | 66 | 0.97 (0.76; 1.24) | 0.792 | 38 | 0.99 (0.72; 1.36) | 0.934 |
| Non-Hodgkin lymphoma | 409,110 | 1,744 | 1.00 (Ref.) | 21 | 0.88 (0.57; 1.36) | 0.564 | 27 | 0.79 (0.54; 1.16) | 0.234 | 19 | 1.00 (0.64; 1.57) | 0.997 |
| Multiple Myeloma | 409,110 | 921 | 1.00 (Ref.) | 13 | 0.97 (0.56; 1.67) | 0.900 | 21 | 1.20 (0.78; 1.85) | 0.406 | 11 | 1.10 (0.60; 1.99) | 0.763 |
| Leukaemia | 409,110 | 1,116 | 1.00 (Ref.) | 16 | 1.09 (0.66; 1.79) | 0.728 | 25 | 1.24 (0.84; 1.85) | 0.281 | 11 | 0.96 (0.53; 1.74) | 0.893 |
| **Model 2** |  |  |  |  | **HR 95% CI** | P value |  | **HR 95% CI** | P value |  | **HR 95% CI** | P value |
| Overall | 409,110 | 38,042 | 1.00 (Ref.) | 463 | 0.85 (0.78; 0.93) | **0.001** | 686 | 0.90 (0.84; 0.97) | **0.008** | 405 | 0.97 (0.88; 1.07) | 0.538 |
| Head & neck | 409,110 | 848 | 1.00 (Ref.) | 12 | 1.00 (0.56; 1.77) | 0.993 | 21 | 1.32 (0.85; 2.03) | 0.217 | 9 | 1.11 (0.58; 2.15) | 0.749 |
| Oesophagus | 409,110 | 1,010 | 1.00 (Ref.) | 11 | 1.05 (0.58; 1.90) | 0.877 | 7 | 0.46 (0.22; 0.97) | 0.043 | 13 | 1.54 (0.89; 2.66) | 0.123 |
| Stomach | 409,110 | 775 | 1.00 (Ref.) | 8 | 0.86 (0.43; 1.74) | 0.679 | 11 | 0.89 (0.49; 1.62) | 0.709 | 7 | 1.00 (0.47; 2.10) | 0.991 |
| Colorectal | 409,110 | 4,679 | 1.00 (Ref.) | 43 | 0.71 (0.52; 0.96) | **0.024** | 75 | 0.87 (0.69; 1.09) | 0.214 | 42 | 0.86 (0.64; 1.17) | 0.348 |
| Colon | 409,110 | 3,340 | 1.00 (Ref.) | 29 | 0.66 (0.46; 0.95) | **0.027** | 52 | 0.83 (0.63; 1.09) | 0.185 | 29 | 0.81 (0.56; 1.17) | 0.264 |
| Proximal | 409,110 | 1,680 | 1.00 (Ref.) | 9 | 0.41 (0.21; 0.78) | **0.007** | 29 | 0.91 (0.63; 1.32) | 0.623 | 15 | 0.80 (0.48; 1.34) | 0.402 |
| Distal | 409,110 | 1,444 | 1.00 (Ref.) | 17 | 0.90 (0.56; 1.45) | 0.661 | 18 | 0.67 (0.42; 1.07) | 0.097 | 15 | 1.03 (0.62; 1.71) | 0.924 |
| Rectum | 409,110 | 2,045 | 1.00 (Ref.) | 17 | 0.70 (0.44; 1.11) | 0.130 | 35 | 0.95 (0.68; 1.33) | 0.768 | 24 | 1.21 (0.81; 1.81) | 0.353 |
| Pancreas | 409,110 | 1,133 | 1.00 (Ref.) | 9 | 0.62 (0.32; 1.20) | 0.159 | 21 | 1.02 (0.66; 1.57) | 0.933 | 6 | 0.50 (0.22; 1.11) | 0.087 |
| Lung | 409,110 | 3,306 | 1.00 (Ref.) | 27 | 0.77 (0.53; 1.12) | 0.176 | 46 | 0.86 (0.65; 1.16) | 0.326 | 28 | 0.83 (0.57; 1.21) | 0.334 |
| Melanoma | 409,110 | 1,979 | 1.00 (Ref.) | 19 | 0.67 (0.43; 1.05) | 0.084 | 28 | 0.67 (0.46; 0.97) | **0.033** | 17 | 0.79 (0.49; 1.27) | 0.334 |
| Breast | 218,391 | 6,895 | 1.00 (Ref.) | 138 | 0.93 (0.78; 1.10) | 0.375 | 194 | 0.87 (0.76; 1.01) | 0.067 | 127 | 1.08 (0.91; 1.29) | 0.385 |
| Premenopausal | 54,775 | 1,776 | 1.00 (Ref.) | 50 | 1.03 (0.78; 1.37) | 0.838 | 52 | 0.80 (0.61; 1.06) | 0.119 | 22 | 0.93 (0.61; 1.42) | 0.734 |
| Postmenopausal | 130,625 | 3,668 | 1.00 (Ref.) | 71 | 0.89 (0.70; 1.12) | 0.317 | 114 | 0.90 (0.74; 1.08) | 0.250 | 79 | 1.07 (0.86; 1.34) | 0.530 |
|  |  |  |  |  |  |  |  |  |  |  |  |  |
| Ovary | 218,391 | 870 | 1.00 (Ref.) | 19 | 1.13 (0.72; 1.79) | 0.597 | 28 | 1.07 (0.73; 1.56) | 0.739 | 18 | 1.19 (0.75; 1.90) | 0.467 |
| Prostate | 190,543 | 7,492 | 1.00 (Ref.) | 47 | 0.58 (0.43; 0.77) | **<0.001** | 82 | 0.90 (0.73; 1.12) | 0.360 | 41 | 0.88 (0.65; 1.20) | 0.428 |
| Kidney | 409,110 | 1,227 | 1.00 (Ref.) | 12 | 0.80 (0.45; 1.41) | 0.439 | 17 | 0.83 (0.51; 1.34) | 0.437 | 12 | 1.04 (0.59; 1.84) | 0.888 |
| Bladder | 409,110 | 2,054 | 1.00 (Ref.) | 18 | 0.88 (0.55; 1.40) | 0.578 | 35 | 1.20 (0.86; 1.67) | 0.293 | 16 | 0.97 (0.59; 1.58) | 0.890 |
| Brain | 409,110 | 760 | 1.00 (Ref.) | 8 | 0.73 (0.36; 1.48) | 0.386 | 12 | 0.78 (0.44; 1.39) | 0.404 | 10 | 1.26 (0.67; 2.35) | 0.475 |
| Haematological | 409,110 | 3,583 | 1.00 (Ref.) | 47 | 0.95 (0.71; 1.27) | 0.749 | 66 | 0.97 (0.76; 1.24) | 0.823 | 38 | 0.99 (0.72; 1.36) | 0.949 |
| Non-Hodgkin lymphoma | 409,110 | 1,744 | 1.00 (Ref.) | 21 | 0.88 (0.57; 1.36) | 0.563 | 27 | 0.80 (0.54; 1.17) | 0.243 | 19 | 1.00 (0.64; 1.57) | 0.999 |
| Multiple Myeloma | 409,110 | 921 | 1.00 (Ref.) | 13 | 0.96 (0.55; 1.67) | 0.888 | 21 | 1.20 (0.78; 1.86) | 0.404 | 11 | 1.09 (0.60; 1.99) | 0.766 |
| Leukaemia | 409,110 | 1,116 | 1.00 (Ref.) | 16 | 1.11 (0.67; 1.82) | 0.693 | 25 | 1.25 (0.84; 1.86) | 0.269 | 11 | 0.97 (0.53; 1.75) | 0.912 |
| **Model 3** |  |  |  |  | **HR 95% CI** | P value |  | **HR 95% CI** | P value |  | **HR 95% CI** | P value |
| Overall | 409,110 | 38,042 | 1.00 (Ref.) | 463 | 0.86 (0.78; 0.94) | **0.001** | 686 | 0.91 (0.84; 0.98) | **0.011** | 405 | 0.97 (0.88; 1.07) | 0.562 |
| Head & neck | 409,110 | 848 | 1.00 (Ref.) | 12 | 1.00 (0.56; 1.77) | 0.999 | 21 | 1.32 (0.86; 2.04) | 0.209 | 9 | 1.11 (0.58; 2.15) | 0.747 |
| Oesophagus | 409,110 | 1,010 | 1.00 (Ref.) | 11 | 1.06 (0.59; 1.93) | 0.838 | 7 | 0.47 (0.23; 1.00) | **0.050** | 13 | 1.55 (0.89; 2.68) | 0.119 |
| Stomach | 409,110 | 775 | 1.00 (Ref.) | 8 | 0.87 (0.43; 1.75) | 0.695 | 11 | 0.90 (0.50; 1.64) | 0.740 | 7 | 1.00 (0.47; 2.11) | 0.998 |
| Colorectal | 409,110 | 4,679 | 1.00 (Ref.) | 43 | 0.71 (0.52; 0.96) | **0.024** | 75 | 0.87 (0.69; 1.09) | 0.217 | 42 | 0.86 (0.64; 1.17) | 0.348 |
| Colon | 409,110 | 3,340 | 1.00 (Ref.) | 29 | 0.66 (0.46; 0.96) | **0.028** | 52 | 0.83 (0.63; 1.10) | 0.194 | 29 | 0.81 (0.56; 1.17) | 0.266 |
| Proximal | 409,110 | 1,680 | 1.00 (Ref.) | 9 | 0.41 (0.21; 0.79) | **0.007** | 29 | 0.92 (0.63; 1.32) | 0.642 | 15 | 0.81 (0.48; 1.34) | 0.406 |
| Distal | 409,110 | 1,444 | 1.00 (Ref.) | 17 | 0.90 (0.56; 1.45) | 0.664 | 18 | 0.67 (0.42; 1.07) | 0.098 | 15 | 1.03 (0.62; 1.71) | 0.922 |
| Rectum | 409,110 | 2,045 | 1.00 (Ref.) | 17 | 0.70 (0.44; 1.11) | 0.128 | 35 | 0.95 (0.68; 1.33) | 0.759 | 24 | 1.21 (0.81; 1.81) | 0.355 |
| Pancreas | 409,110 | 1,133 | 1.00 (Ref.) | 9 | 0.63 (0.33; 1.21) | 0.167 | 21 | 1.03 (0.67; 1.59) | 0.881 | 6 | 0.50 (0.22; 1.11) | 0.090 |
| Lung | 409,110 | 3,306 | 1.00 (Ref.) | 27 | 0.78 (0.53; 1.14) | 0.202 | 46 | 0.88 (0.66; 1.18) | 0.411 | 28 | 0.84 (0.58; 1.22) | 0.352 |
| Melanoma | 409,110 | 1,979 | 1.00 (Ref.) | 19 | 0.67 (0.43; 1.06) | 0.084 | 28 | 0.67 (0.46; 0.97) | **0.034** | 17 | 0.79 (0.49; 1.27) | 0.334 |
| Breast | 218,391 | 6,895 | 1.00 (Ref.) | 138 | 0.93 (0.78; 1.10) | 0.375 | 194 | 0.87 (0.76; 1.01) | 0.067 | 127 | 1.08 (0.91; 1.29) | 0.385 |
| Premenopausal | 54,775 | 1,776 | 1.00 (Ref.) | 50 | 1.01 (0.76; 1.33) | 0.970 | 52 | 0.78 (0.59; 1.03) | 0.082 | 22 | 0.91 (0.60; 1.39) | 0.657 |
| Postmenopausal | 130,625 | 3,668 | 1.00 (Ref.) | 71 | 0.88 (0.70; 1.12) | 0.300 | 113 | 0.88 (0.73; 1.06) | 0.186 | 79 | 1.06 (0.85; 1.33) | 0.604 |
| Uterine | 218,391 | 1,131 | 1.00 (Ref.) | 23 | 0.97 (0.64; 1.46) | 0.870 | 30 | 0.88 (0.61; 1.27) | 0.493 | 18 | 0.90 (0.56; 1.43) | 0.649 |
| Ovary | 218,391 | 870 | 1.00 (Ref.) | 19 | 1.13 (0.72; 1.79) | 0.594 | 28 | 1.07 (0.73; 1.56) | 0.734 | 18 | 1.19 (0.75; 1.90) | 0.465 |
| Prostate | 190,543 | 7,492 | 1.00 (Ref.) | 47 | 0.58 (0.43; 0.77) | **<0.001** | 82 | 0.90 (0.72; 1.12) | 0.348 | 41 | 0.88 (0.65; 1.20) | 0.427 |
| Kidney | 409,110 | 1,227 | 1.00 (Ref.) | 12 | 0.81 (0.46; 1.44) | 0.472 | 17 | 0.85 (0.52; 1.37) | 0.502 | 12 | 1.05 (0.59; 1.85) | 0.870 |
| Bladder | 409,110 | 2,054 | 1.00 (Ref.) | 18 | 0.89 (0.56; 1.41) | 0.608 | 35 | 1.21 (0.87; 1.70) | 0.256 | 16 | 0.97 (0.59; 1.59) | 0.901 |
| Brain | 409,110 | 760 | 1.00 (Ref.) | 8 | 0.73 (0.36; 1.48) | 0.386 | 12 | 0.78 (0.44; 1.39) | 0.405 | 10 | 1.26 (0.67; 2.35) | 0.475 |
| Haematological | 409,110 | 3,583 | 1.00 (Ref.) | 47 | 0.96 (0.72; 1.28) | 0.765 | 66 | 0.98 (0.77; 1.25) | 0.855 | 38 | 0.99 (0.72; 1.37) | 0.957 |
| Non-Hodgkin lymphoma | 409,110 | 1,744 | 1.00 (Ref.) | 21 | 0.88 (0.57; 1.36) | 0.576 | 27 | 0.80 (0.55; 1.17) | 0.257 | 19 | 1.00 (0.64; 1.58) | 0.994 |
| Multiple Myeloma | 409,110 | 921 | 1.00 (Ref.) | 13 | 0.96 (0.55; 1.67) | 0.887 | 21 | 1.20 (0.78; 1.86) | 0.405 | 11 | 1.09 (0.60; 1.99) | 0.767 |
| Leukaemia | 409,110 | 1,116 | 1.00 (Ref.) | 16 | 1.11 (0.67; 1.82) | 0.683 | 25 | 1.26 (0.85; 1.87) | 0.258 | 11 | 0.97 (0.53; 1.76) | 0.916 |

Data presented as adjusted hazard ratio (HR) and its 95% confidence interval (95% CI) by type of diets. Meat-eaters were used as the reference group.

"model 1" (minimally adjusted) included sociodemographic covariates (age, sex, deprivation, and ethnicity); "model 2" additionally included lifestyle factors (smoking, alcohol intake and total physical activity); and "model 3" included model 2 plus multimorbidity.

Table S5: Landmark sensitivity analysis: Association between types of diet and cancer incidence for all Models after excluding events in the first 2 years of follow-up.

| **Cancer site** | **Total** | **Event** | **Meat-eaters** | **Event** | **Vegan & Vegetarian** |  | **Event** | **Pescatarian** |  | **Event** | **Fish & Poultry** |  |
| --- | --- | --- | --- | --- | --- | --- | --- | --- | --- | --- | --- | --- |
| **Model 0** |  |  |  |  | **HR 95% CI** | P value |  | **HR 95% CI** | P value |  | **HR 95% CI** | P value |
| Overall | 403805 | 32960 | 1.00 (Ref.) | 405 | 0.65 (0.59; 0.71) | **<0.001** | 578 | 0.71 (0.65; 0.77) | **<0.001** | 348 | 0.89 (0.80; 0.98) | **0.025** |
| Head & neck | 408996 | 739 | 1.00 (Ref.) | 9 | 0.65 (0.34; 1.26) | 0.204 | 21 | 1.16 (0.75; 1.79) | 0.501 | 7 | 0.80 (0.38; 1.68) | 0.555 |
| Oesophagus | 408980 | 887 | 1.00 (Ref.) | 10 | 0.61 (0.32; 1.13) | 0.115 | 5 | 0.23 (0.10; 0.55) | **0.001** | 9 | 0.86 (0.45; 1.65) | 0.648 |
| Stomach | 409013 | 686 | 1.00 (Ref.) | 4 | 0.31 (0.12; 0.84) | **0.020** | 8 | 0.48 (0.24; 0.96) | **0.037** | 6 | 0.74 (0.33; 1.65) | 0.458 |
| Colorectal | 408405 | 3996 | 1.00 (Ref.) | 39 | 0.52 (0.38; 0.72) | **<0.001** | 61 | 0.62 (0.48; 0.80) | **<0.001** | 38 | 0.80 (0.58; 1.10) | 0.172 |
| Colon | 408604 | 2847 | 1.00 (Ref.) | 27 | 0.51 (0.35; 0.74) | **<0.001** | 43 | 0.62 (0.46; 0.83) | **0.002** | 27 | 0.80 (0.55; 1.17) | 0.246 |
| Proximal | 408900 | 1473 | 1.00 (Ref.) | 9 | 0.33 (0.17; 0.63) | **0.001** | 26 | 0.72 (0.49; 1.06) | 0.097 | 15 | 0.86 (0.52; 1.43) | 0.557 |
| Distal | 408872 | 1213 | 1.00 (Ref.) | 16 | 0.71 (0.43; 1.16) | 0.170 | 14 | 0.47 (0.28; 0.80) | **0.005** | 13 | 0.90 (0.52; 1.56) | 0.717 |
| Rectum | 408833 | 1779 | 1.00 (Ref.) | 16 | 0.48 (0.29; 0.79) | **0.004** | 28 | 0.64 (0.44; 0.93) | **0.020** | 22 | 1.04 (0.68; 1.59) | 0.845 |
| Pancreas | 409002 | 1032 | 1.00 (Ref.) | 6 | 0.31 (0.14; 0.70) | **0.004** | 17 | 0.67 (0.42; 1.09) | 0.105 | 6 | 0.49 (0.22; 1.09) | 0.082 |
| Lung | 408732 | 2941 | 1.00 (Ref.) | 24 | 0.44 (0.29; 0.65) | **<0.001** | 39 | 0.54 (0.39; 0.74) | **<0.001** | 25 | 0.72 (0.48; 1.06) | 0.097 |
| Melanoma | 408857 | 1735 | 1.00 (Ref.) | 17 | 0.53 (0.33; 0.85) | **0.008** | 24 | 0.56 (0.38; 0.84) | **0.005** | 14 | 0.68 (0.40; 1.15) | 0.151 |
| Breast | 217207 | 5794 | 1.00 (Ref.) | 115 | 0.85 (0.70; 1.02) | 0.078 | 160 | 0.83 (0.71; 0.97) | **0.018** | 105 | 1.07 (0.88; 1.29) | 0.510 |
| Premenopausal | 54,590 | 1,240 | 1.00 (Ref.) | 41 | 0.93 (0.68; 1.27) | 0.647 | 41 | 0.72 (0.53; 0.99) | 0.040 | 20 | 0.96 (0.62; 1.49) | 0.860 |
| Postmenopausal | 129,922 | 3,656 | 1.00 (Ref.) | 57 | 0.78 (0.60; 1.01) | 0.063 | 96 | 0.87 (0.71; 1.07) | 0.182 | 63 | 1.00 (0.78; 1.28) | 0.987 |
| Uterine | 218222 | 970 | 1.00 (Ref.) | 21 | 0.93 (0.60; 1.43) | 0.734 | 28 | 0.87 (0.59; 1.26) | 0.453 | 15 | 0.91 (0.55; 1.52) | 0.717 |
| Ovary | 218251 | 739 | 1.00 (Ref.) | 17 | 0.99 (0.61; 1.59) | 0.952 | 23 | 0.93 (0.62; 1.42) | 0.749 | 16 | 1.27 (0.78; 2.09) | 0.337 |
| Prostate | 189754 | 6721 | 1.00 (Ref.) | 44 | 0.48 (0.36; 0.64) | **<0.001** | 70 | 0.70 (0.55; 0.89) | **0.003** | 38 | 0.91 (0.66; 1.25) | 0.570 |
| Kidney | 408969 | 1088 | 1.00 (Ref.) | 12 | 0.59 (0.33; 1.04) | 0.070 | 15 | 0.56 (0.34; 0.94) | **0.027** | 12 | 0.93 (0.53; 1.64) | 0.803 |
| Bladder | 408832 | 1783 | 1.00 (Ref.) | 17 | 0.51 (0.32; 0.82) | **0.006** | 32 | 0.73 (0.52; 1.04) | 0.081 | 13 | 0.61 (0.36; 1.06) | 0.080 |
| Brain | 408996 | 650 | 1.00 (Ref.) | 7 | 0.58 (0.27; 1.22) | 0.148 | 10 | 0.63 (0.34; 1.17) | 0.145 | 9 | 1.17 (0.60; 2.25) | 0.644 |
| Haematological | 408676 | 3162 | 1.00 (Ref.) | 44 | 0.75 (0.55; 1.00) | 0.053 | 59 | 0.76 (0.59; 0.98) | **0.038** | 35 | 0.93 (0.67; 1.30) | 0.684 |
| Non-Hodgkin lymphoma | 408877 | 1522 | 1.00 (Ref.) | 18 | 0.63 (0.40; 1.01) | 0.055 | 21 | 0.56 (0.37; 0.87) | **0.009** | 17 | 0.94 (0.58; 1.52) | 0.807 |
| Multiple Myeloma | 409022 | 835 | 1.00 (Ref.) | 12 | 0.77 (0.44; 1.36) | 0.371 | 20 | 0.98 (0.63; 1.52) | 0.921 | 11 | 1.11 (0.61; 2.01) | 0.729 |
| Leukaemia | 408994 | 1002 | 1.00 (Ref.) | 16 | 0.86 (0.52; 1.40) | 0.537 | 24 | 0.98 (0.65; 1.47) | 0.913 | 10 | 0.84 (0.45; 1.57) | 0.588 |
| **Model 1** |  |  |  |  | **HR 95% CI** | P value |  | **HR 95% CI** | P value |  | **HR 95% CI** | P value |
| Overall | 403805 | 32960 | 1.00 (Ref.) | 405 | 0.84 (0.76; 0.92) | **<0.001** | 578 | 0.86 (0.79; 0.93) | **<0.001** | 348 | 0.95 (0.85; 1.05) | 0.317 |
| Head & neck | 408996 | 739 | 1.00 (Ref.) | 9 | 0.77 (0.40; 1.50) | 0.444 | 21 | 1.39 (0.90; 2.15) | 0.139 | 7 | 0.92 (0.43; 1.93) | 0.816 |
| Oesophagus | 408980 | 887 | 1.00 (Ref.) | 10 | 0.99 (0.53; 1.85) | 0.970 | 5 | 0.34 (0.14; 0.83) | **0.018** | 9 | 1.12 (0.58; 2.16) | 0.734 |
| Stomach | 409013 | 686 | 1.00 (Ref.) | 4 | 0.46 (0.17; 1.22) | 0.119 | 8 | 0.69 (0.35; 1.40) | 0.305 | 6 | 0.92 (0.41; 2.05) | 0.829 |
| Colorectal | 408405 | 3996 | 1.00 (Ref.) | 39 | 0.73 (0.53; 1.00) | 0.052 | 61 | 0.80 (0.62; 1.03) | 0.088 | 38 | 0.90 (0.65; 1.24) | 0.519 |
| Colon | 408604 | 2847 | 1.00 (Ref.) | 27 | 0.70 (0.48; 1.03) | 0.070 | 43 | 0.78 (0.58; 1.06) | 0.114 | 27 | 0.87 (0.60; 1.28) | 0.488 |
| Proximal | 408900 | 1473 | 1.00 (Ref.) | 9 | 0.45 (0.24; 0.88) | **0.018** | 26 | 0.91 (0.61; 1.34) | 0.615 | 15 | 0.90 (0.54; 1.50) | 0.698 |
| Distal | 408872 | 1213 | 1.00 (Ref.) | 16 | 0.98 (0.60; 1.61) | 0.932 | 14 | 0.61 (0.36; 1.03) | 0.067 | 13 | 1.05 (0.61; 1.81) | 0.872 |
| Rectum | 408833 | 1779 | 1.00 (Ref.) | 16 | 0.69 (0.42; 1.14) | 0.146 | 28 | 0.86 (0.59; 1.24) | 0.417 | 22 | 1.25 (0.82; 1.90) | 0.303 |
| Pancreas | 409002 | 1032 | 1.00 (Ref.) | 6 | 0.44 (0.20; 0.98) | **0.045** | 17 | 0.88 (0.54; 1.42) | 0.594 | 6 | 0.53 (0.24; 1.18) | 0.122 |
| Lung | 408732 | 2941 | 1.00 (Ref.) | 24 | 0.61 (0.41; 0.92) | **0.018** | 39 | 0.68 (0.49; 0.93) | **0.016** | 25 | 0.70 (0.47; 1.03) | 0.072 |
| Melanoma | 408857 | 1735 | 1.00 (Ref.) | 17 | 0.70 (0.43; 1.12) | 0.137 | 24 | 0.66 (0.44; 0.99) | **0.045** | 14 | 0.76 (0.45; 1.28) | 0.296 |
| Breast | 217207 | 5794 | 1.00 (Ref.) | 115 | 0.91 (0.76; 1.09) | 0.315 | 160 | 0.86 (0.73; 1.01) | 0.060 | 105 | 1.07 (0.89; 1.30) | 0.469 |
| Premenopausal | 54,590 | 1,240 | 1.00 (Ref.) | 41 | 0.97 (0.71; 1.32) | 0.823 | 41 | 0.73 (0.54; 1.00) | 0.052 | 20 | 0.97 (0.63; 1.52) | 0.908 |
| Postmenopausal | 129,922 | 3,656 | 1.00 (Ref.) | 57 | 0.82 (0.63; 1.06) | 0.133 | 96 | 0.89 (0.73; 1.09) | 0.275 | 63 | 1.00 (0.78; 1.29) | 0.972 |
| Uterine | 218222 | 970 | 1.00 (Ref.) | 21 | 1.09 (0.71; 1.69) | 0.695 | 28 | 0.97 (0.67; 1.42) | 0.885 | 15 | 0.91 (0.54; 1.51) | 0.706 |
| Ovary | 218251 | 739 | 1.00 (Ref.) | 17 | 1.21 (0.75; 1.97) | 0.433 | 23 | 1.07 (0.70; 1.62) | 0.758 | 16 | 1.28 (0.78; 2.11) | 0.324 |
| Prostate | 189754 | 6721 | 1.00 (Ref.) | 44 | 0.61 (0.45; 0.82) | **0.001** | 70 | 0.87 (0.69; 1.10) | 0.239 | 38 | 0.93 (0.68; 1.28) | 0.651 |
| Kidney | 408969 | 1088 | 1.00 (Ref.) | 12 | 0.86 (0.48; 1.52) | 0.599 | 15 | 0.79 (0.47; 1.31) | 0.358 | 12 | 1.14 (0.64; 2.01) | 0.661 |
| Bladder | 408832 | 1783 | 1.00 (Ref.) | 17 | 0.88 (0.55; 1.42) | 0.607 | 32 | 1.18 (0.83; 1.68) | 0.350 | 13 | 0.85 (0.49; 1.46) | 0.550 |
| Brain | 408996 | 650 | 1.00 (Ref.) | 7 | 0.75 (0.36; 1.59) | 0.456 | 10 | 0.76 (0.41; 1.42) | 0.393 | 9 | 1.32 (0.68; 2.55) | 0.408 |
| Haematological | 408676 | 3162 | 1.00 (Ref.) | 44 | 1.01 (0.75; 1.37) | 0.932 | 59 | 0.98 (0.76; 1.27) | 0.888 | 35 | 1.03 (0.74; 1.44) | 0.855 |
| Non-Hodgkin lymphoma | 408877 | 1522 | 1.00 (Ref.) | 18 | 0.87 (0.55; 1.39) | 0.563 | 21 | 0.71 (0.46; 1.09) | 0.118 | 17 | 1.03 (0.64; 1.66) | 0.918 |
| Multiple Myeloma | 409022 | 835 | 1.00 (Ref.) | 12 | 0.99 (0.56; 1.76) | 0.978 | 20 | 1.26 (0.81; 1.97) | 0.307 | 11 | 1.22 (0.67; 2.21) | 0.520 |
| Leukaemia | 408994 | 1002 | 1.00 (Ref.) | 16 | 1.21 (0.74; 1.99) | 0.447 | 24 | 1.34 (0.89; 2.00) | 0.163 | 10 | 0.97 (0.52; 1.81) | 0.928 |
| **Model 2** |  |  |  |  | **HR 95% CI** | P value |  | **HR 95% CI** | P value |  | **HR 95% CI** | P value |
| Overall | 403805 | 32960 | 1.00 (Ref.) | 405 | 0.86 (0.78; 0.95) | **0.002** | 578 | 0.88 (0.81; 0.95) | **0.002** | 348 | 0.97 (0.87; 1.07) | 0.526 |
| Head & neck | 408996 | 739 | 1.00 (Ref.) | 9 | 0.85 (0.44; 1.65) | 0.631 | 21 | 1.50 (0.97; 2.32) | 0.067 | 7 | 0.98 (0.47; 2.08) | 0.967 |
| Oesophagus | 408980 | 887 | 1.00 (Ref.) | 10 | 1.07 (0.57; 2.00) | 0.838 | 5 | 0.37 (0.15; 0.90) | **0.028** | 9 | 1.21 (0.63; 2.33) | 0.575 |
| Stomach | 409013 | 686 | 1.00 (Ref.) | 4 | 0.48 (0.18; 1.27) | 0.139 | 8 | 0.72 (0.36; 1.45) | 0.364 | 6 | 0.95 (0.42; 2.13) | 0.901 |
| Colorectal | 408405 | 3996 | 1.00 (Ref.) | 39 | 0.74 (0.54; 1.02) | 0.065 | 61 | 0.81 (0.63; 1.05) | 0.108 | 38 | 0.91 (0.66; 1.25) | 0.565 |
| Colon | 408604 | 2847 | 1.00 (Ref.) | 27 | 0.71 (0.49; 1.04) | 0.082 | 43 | 0.79 (0.59; 1.07) | 0.136 | 27 | 0.88 (0.60; 1.29) | 0.520 |
| Proximal | 408900 | 1473 | 1.00 (Ref.) | 9 | 0.46 (0.24; 0.89) | **0.020** | 26 | 0.92 (0.62; 1.36) | 0.678 | 15 | 0.91 (0.55; 1.52) | 0.729 |
| Distal | 408872 | 1213 | 1.00 (Ref.) | 16 | 0.99 (0.60; 1.63) | 0.974 | 14 | 0.61 (0.36; 1.04) | 0.069 | 13 | 1.06 (0.61; 1.82) | 0.848 |
| Rectum | 408833 | 1779 | 1.00 (Ref.) | 16 | 0.71 (0.43; 1.16) | 0.167 | 28 | 0.86 (0.59; 1.26) | 0.447 | 22 | 1.27 (0.83; 1.93) | 0.274 |
| Pancreas | 409002 | 1032 | 1.00 (Ref.) | 6 | 0.45 (0.20; 1.02) | 0.055 | 17 | 0.90 (0.56; 1.45) | 0.661 | 6 | 0.55 (0.24; 1.22) | 0.139 |
| Lung | 408732 | 2941 | 1.00 (Ref.) | 24 | 0.76 (0.51; 1.14) | 0.186 | 39 | 0.82 (0.59; 1.12) | 0.209 | 25 | 0.83 (0.56; 1.23) | 0.354 |
| Melanoma | 408857 | 1735 | 1.00 (Ref.) | 17 | 0.69 (0.43; 1.11) | 0.124 | 24 | 0.65 (0.44; 0.98) | **0.039** | 14 | 0.75 (0.44; 1.26) | 0.276 |
| Breast | 217207 | 5794 | 1.00 (Ref.) | 115 | 0.90 (0.75; 1.09) | 0.286 | 159 | 0.85 (0.72; 0.99) | **0.037** | 5 | 1.06 (0.88; 1.29) | 0.535 |
| Premenopausal | 54,590 | 1,240 | 1.00 (Ref.) |  |  |  |  |  |  |  |  |  |
| Postmenopausal | 129,922 | 3,656 | 1.00 (Ref.) |  |  |  |  |  |  |  |  |  |
| Uterine | 218222 | 970 | 1.00 (Ref.) | 21 | 1.02 (0.66; 1.58) | 0.916 | 28 | 0.95 (0.65; 1.39) | 0.799 | 15 | 0.87 (0.52; 1.46) | 0.606 |
| Ovary | 218251 | 739 | 1.00 (Ref.) | 17 | 1.18 (0.73; 1.91) | 0.508 | 23 | 1.03 (0.68; 1.56) | 0.893 | 16 | 1.24 (0.76; 2.04) | 0.388 |
| Prostate | 189754 | 6721 | 1.00 (Ref.) | 44 | 0.60 (0.44; 0.81) | **0.001** | 70 | 0.85 (0.67; 1.08) | 0.188 | 38 | 0.92 (0.67; 1.26) | 0.587 |
| Kidney | 408969 | 1088 | 1.00 (Ref.) | 12 | 0.89 (0.50; 1.58) | 0.701 | 15 | 0.82 (0.49; 1.37) | 0.447 | 12 | 1.17 (0.66; 2.07) | 0.586 |
| Bladder | 408832 | 1783 | 1.00 (Ref.) | 17 | 0.95 (0.59; 1.53) | 0.819 | 32 | 1.25 (0.88; 1.78) | 0.208 | 13 | 0.90 (0.52; 1.56) | 0.712 |
| Brain | 408996 | 650 | 1.00 (Ref.) | 7 | 0.75 (0.35; 1.58) | 0.444 | 10 | 0.75 (0.40; 1.41) | 0.371 | 9 | 1.31 (0.68; 2.54) | 0.419 |
| Thyroid | 409060 | 241 | 1.00 (Ref.) | 7 | 1.40 (0.66; 2.98) | 0.386 | 3 | 0.44 (0.14; 1.37) | 0.155 | 3 | 0.87 (0.28; 2.71) | 0.805 |
| Haematological | 408676 | 3162 | 1.00 (Ref.) | 44 | 1.02 (0.75; 1.37) | 0.916 | 59 | 0.99 (0.76; 1.28) | 0.914 | 35 | 1.03 (0.74; 1.44) | 0.844 |
| Non-Hodgkin lymphoma | 408877 | 1522 | 1.00 (Ref.) | 18 | 0.87 (0.55; 1.39) | 0.559 | 21 | 0.71 (0.46; 1.10) | 0.122 | 17 | 1.03 (0.63; 1.65) | 0.919 |
| Multiple Myeloma | 409022 | 835 | 1.00 (Ref.) | 12 | 0.99 (0.56; 1.75) | 0.967 | 20 | 1.26 (0.81; 1.97) | 0.307 | 11 | 1.21 (0.67; 2.20) | 0.523 |
| Leukaemia | 408994 | 1002 | 1.00 (Ref.) | 16 | 1.23 (0.75; 2.02) | 0.417 | 24 | 1.34 (0.89; 2.01) | 0.157 | 10 | 0.98 (0.52; 1.83) | 0.946 |
| **Model 3** |  |  |  |  | **HR 95% CI** | P value |  | **HR 95% CI** | P value |  | **HR 95% CI** | P value |
| Overall | 403805 | 32960 | 1.00 (Ref.) | 405 | 0.86 (0.78; 0.95) | **0.003** | 578 | 0.88 (0.81; 0.96) | **0.002** | 348 | 0.97 (0.87; 1.08) | 0.546 |
| Head & neck | 408996 | 739 | 1.00 (Ref.) | 9 | 0.85 (0.44; 1.65) | 0.634 | 21 | 1.51 (0.97; 2.33) | 0.066 | 7 | 0.98 (0.47; 2.08) | 0.968 |
| Oesophagus | 408980 | 887 | 1.00 (Ref.) | 10 | 1.08 (0.58; 2.03) | 0.799 | 5 | 0.38 (0.16; 0.92) | **0.032** | 9 | 1.21 (0.63; 2.34) | 0.564 |
| Stomach | 409013 | 686 | 1.00 (Ref.) | 4 | 0.48 (0.18; 1.28) | 0.143 | 8 | 0.73 (0.36; 1.47) | 0.380 | 6 | 0.95 (0.43; 2.13) | 0.906 |
| Colorectal | 408405 | 3996 | 1.00 (Ref.) | 39 | 0.74 (0.54; 1.02) | 0.064 | 61 | 0.81 (0.63; 1.05) | 0.108 | 38 | 0.91 (0.66; 1.25) | 0.565 |
| Colon | 408604 | 2847 | 1.00 (Ref.) | 27 | 0.71 (0.49; 1.05) | 0.084 | 43 | 0.80 (0.59; 1.08) | 0.140 | 27 | 0.88 (0.60; 1.29) | 0.523 |
| Proximal | 408900 | 1473 | 1.00 (Ref.) | 9 | 0.46 (0.24; 0.89) | **0.021** | 26 | 0.92 (0.63; 1.36) | 0.690 | 15 | 0.91 (0.55; 1.52) | 0.732 |
| Distal | 408872 | 1213 | 1.00 (Ref.) | 16 | 0.99 (0.60; 1.63) | 0.975 | 14 | 0.61 (0.36; 1.04) | 0.070 | 13 | 1.06 (0.61; 1.82) | 0.847 |
| Rectum | 408833 | 1779 | 1.00 (Ref.) | 16 | 0.70 (0.43; 1.15) | 0.164 | 28 | 0.86 (0.59; 1.25) | 0.438 | 22 | 1.26 (0.83; 1.93) | 0.276 |
| Pancreas | 409002 | 1032 | 1.00 (Ref.) | 6 | 0.46 (0.21; 1.03) | 0.058 | 17 | 0.91 (0.56; 1.48) | 0.709 | 6 | 0.55 (0.25; 1.22) | 0.143 |
| Lung | 408732 | 2941 | 1.00 (Ref.) | 24 | 0.77 (0.52; 1.16) | 0.211 | 39 | 0.84 (0.61; 1.15) | 0.267 | 25 | 0.84 (0.56; 1.24) | 0.372 |
| Melanoma | 408857 | 1735 | 1.00 (Ref.) | 17 | 0.69 (0.43; 1.11) | 0.124 | 24 | 0.65 (0.44; 0.98) | **0.039** | 14 | 0.75 (0.44; 1.26) | 0.277 |
| Breast | 217207 | 5794 | 1.00 (Ref.) | 115 | 0.90 (0.75; 1.09) | 0.287 | 159 | 0.85 (0.72; 0.99) | **0.037** | 105 | 1.06 (0.88; 1.29) | 0.534 |
| Premenopausal | 54,590 | 1,240 | 1.00 (Ref.) | 41 | 0.97 (0.71; 1.32) | 0.837 | 41 | 0.73 (0.53; 0.99) | 0.046 | 20 | 0.98 (0.63; 1.52) | 0.912 |
| Postmenopausal | 129,922 | 3,656 | 1.00 (Ref.) | 57 | 0.84 (0.65; 1.10) | 0.203 | 96 | 0.90 (0.74; 1.10) | 0.311 | 63 | 1.03 (0.80; 1.32) | 0.819 |
| Uterine | 218222 | 970 | 1.00 (Ref.) | 21 | 1.03 (0.67; 1.59) | 0.898 | 28 | 0.96 (0.66; 1.40) | 0.835 | 15 | 0.88 (0.53; 1.46) | 0.615 |
| Ovary | 218251 | 739 | 1.00 (Ref.) | 17 | 1.18 (0.73; 1.91) | 0.506 | 23 | 1.03 (0.68; 1.56) | 0.889 | 16 | 1.24 (0.76; 2.04) | 0.387 |
| Prostate | 189754 | 6721 | 1.00 (Ref.) | 44 | 0.60 (0.44; 0.80) | **0.001** | 70 | 0.85 (0.67; 1.08) | 0.179 | 38 | 0.91 (0.66; 1.26) | 0.585 |
| Kidney | 408969 | 1088 | 1.00 (Ref.) | 12 | 0.91 (0.51; 1.61) | 0.741 | 15 | 0.84 (0.50; 1.40) | 0.508 | 12 | 1.18 (0.67; 2.09) | 0.571 |
| Bladder | 408832 | 1783 | 1.00 (Ref.) | 17 | 0.95 (0.59; 1.54) | 0.846 | 32 | 1.27 (0.89; 1.80) | 0.184 | 13 | 0.91 (0.52; 1.56) | 0.721 |
| Brain | 408996 | 650 | 1.00 (Ref.) | 7 | 0.75 (0.35; 1.58) | 0.443 | 10 | 0.75 (0.40; 1.40) | 0.369 | 9 | 1.31 (0.68; 2.54) | 0.420 |
| Haematological | 408676 | 3162 | 1.00 (Ref.) | 44 | 1.02 (0.76; 1.38) | 0.898 | 59 | 0.99 (0.77; 1.28) | 0.947 | 35 | 1.04 (0.74; 1.45) | 0.836 |
| Non-Hodgkin lymphoma | 408877 | 1522 | 1.00 (Ref.) | 18 | 0.87 (0.55; 1.39) | 0.572 | 21 | 0.72 (0.47; 1.10) | 0.131 | 17 | 1.03 (0.64; 1.66) | 0.912 |
| Multiple Myeloma | 409022 | 835 | 1.00 (Ref.) | 12 | 0.99 (0.56; 1.75) | 0.967 | 20 | 1.26 (0.81; 1.97) | 0.306 | 11 | 1.21 (0.67; 2.21) | 0.523 |
| Leukaemia | 408994 | 1002 | 1.00 (Ref.) | 16 | 1.23 (0.75; 2.03) | 0.410 | 24 | 1.35 (0.90; 2.02) | 0.150 | 10 | 0.98 (0.53; 1.83) | 0.950 |
| **Model 4** |  |  |  |  | **HR 95% CI** | P value |  | **HR 95% CI** | P value |  | **HR 95% CI** | P value |
| Overall | 403805 | 32960 | 1.00 (Ref.) | 405 | 0.88 (0.80; 0.97) | **0.009** | 578 | 0.90 (0.83; 0.98) | **0.013** | 348 | 0.99 (0.89; 1.10) | 0.827 |
| Head & neck | 408996 | 739 | 1.00 (Ref.) | 9 | 0.80 (0.41; 1.55) | 0.511 | 21 | 1.41 (0.91; 2.19) | 0.122 | 7 | 0.93 (0.44; 1.96) | 0.847 |
| Oesophagus | 408980 | 887 | 1.00 (Ref.) | 10 | 1.16 (0.62; 2.17) | 0.641 | 5 | 0.41 (0.17; 0.99) | **0.048** | 9 | 1.30 (0.67; 2.50) | 0.440 |
| Stomach | 409013 | 686 | 1.00 (Ref.) | 4 | 0.52 (0.19; 1.40) | 0.197 | 8 | 0.81 (0.40; 1.62) | 0.543 | 6 | 1.04 (0.46; 2.33) | 0.926 |
| Colorectal | 408405 | 3996 | 1.00 (Ref.) | 39 | 0.77 (0.56; 1.06) | 0.106 | 61 | 0.85 (0.66; 1.09) | 0.197 | 38 | 0.94 (0.69; 1.30) | 0.727 |
| Colon | 408604 | 2847 | 1.00 (Ref.) | 27 | 0.75 (0.51; 1.09) | 0.131 | 43 | 0.83 (0.62; 1.13) | 0.240 | 27 | 0.92 (0.63; 1.35) | 0.673 |
| Proximal | 408900 | 1473 | 1.00 (Ref.) | 9 | 0.48 (0.25; 0.93) | **0.031** | 26 | 0.98 (0.66; 1.44) | 0.910 | 15 | 0.96 (0.58; 1.60) | 0.886 |
| Distal | 408872 | 1213 | 1.00 (Ref.) | 16 | 1.03 (0.63; 1.70) | 0.895 | 14 | 0.64 (0.38; 1.09) | 0.100 | 13 | 1.10 (0.64; 1.90) | 0.735 |
| Rectum | 408833 | 1779 | 1.00 (Ref.) | 16 | 0.73 (0.44; 1.19) | 0.209 | 28 | 0.89 (0.61; 1.30) | 0.559 | 22 | 1.31 (0.86; 1.99) | 0.214 |
| Pancreas | 409002 | 1032 | 1.00 (Ref.) | 6 | 0.48 (0.21; 1.06) | 0.071 | 17 | 0.95 (0.59; 1.53) | 0.829 | 6 | 0.57 (0.25; 1.27) | 0.167 |
| Lung | 408732 | 2941 | 1.00 (Ref.) | 24 | 0.75 (0.50; 1.12) | 0.164 | 39 | 0.81 (0.59; 1.11) | 0.196 | 25 | 0.81 (0.55; 1.21) | 0.307 |
| Melanoma | 408857 | 1735 | 1.00 (Ref.) | 17 | 0.70 (0.43; 1.13) | 0.143 | 24 | 0.67 (0.44; 1.00) | **0.049** | 14 | 0.76 (0.45; 1.29) | 0.307 |
| Breast | 217207 | 5794 | 1.00 (Ref.) | 115 | 0.93 (0.77; 1.12) | 0.423 | 159 | 0.87 (0.74; 1.02) | 0.084 | 105 | 1.09 (0.90; 1.32) | 0.375 |
| Premenopausal | 54,590 | 1,240 | 1.00 (Ref.) | 41 | 0.94 (0.69; 1.29) | 0.701 | 41 | 0.71 (0.52; 0.97) | 0.030 | 20 | 0.95 (0.61; 1.48) | 0.820 |
| Postmenopausal | 129,922 | 3,656 | 1.00 (Ref.) | 57 | 0.87 (0.67; 1.14) | 0.318 | 95 | 0.92 (0.75; 1.13) | 0.439 | 63 | 1.06 (0.83; 1.36) | 0.644 |
| Uterine | 218222 | 970 | 1.00 (Ref.) | 21 | 1.23 (0.79; 1.89) | 0.360 | 28 | 1.20 (0.82; 1.74) | 0.354 | 15 | 1.06 (0.64; 1.76) | 0.827 |
| Ovary | 218251 | 739 | 1.00 (Ref.) | 17 | 1.18 (0.73; 1.92) | 0.494 | 23 | 1.04 (0.68; 1.57) | 0.870 | 16 | 1.25 (0.76; 2.05) | 0.377 |
| Prostate | 189754 | 6721 | 1.00 (Ref.) | 44 | 0.59 (0.44; 0.79) | **<0.001** | 70 | 0.84 (0.66; 1.06) | 0.140 | 38 | 0.90 (0.65; 1.24) | 0.522 |
| Kidney | 408969 | 1088 | 1.00 (Ref.) | 12 | 0.99 (0.56; 1.76) | 0.975 | 15 | 0.93 (0.56; 1.55) | 0.773 | 12 | 1.29 (0.73; 2.28) | 0.382 |
| Bladder | 408832 | 1783 | 1.00 (Ref.) | 17 | 0.98 (0.61; 1.58) | 0.930 | 32 | 1.30 (0.92; 1.85) | 0.139 | 13 | 0.93 (0.54; 1.60) | 0.788 |
| Brain | 408996 | 650 | 1.00 (Ref.) | 7 | 0.74 (0.35; 1.57) | 0.437 | 10 | 0.75 (0.40; 1.40) | 0.363 | 9 | 1.31 (0.68; 2.53) | 0.426 |
| Haematological | 408676 | 3162 | 1.00 (Ref.) | 44 | 1.04 (0.77; 1.41) | 0.776 | 59 | 1.02 (0.79; 1.32) | 0.895 | 35 | 1.06 (0.76; 1.48) | 0.730 |
| Non-Hodgkin lymphoma | 408877 | 1522 | 1.00 (Ref.) | 18 | 0.88 (0.55; 1.41) | 0.606 | 21 | 0.73 (0.47; 1.12) | 0.147 | 17 | 1.04 (0.64; 1.68) | 0.874 |
| Multiple Myeloma | 409022 | 835 | 1.00 (Ref.) | 12 | 1.02 (0.57; 1.81) | 0.949 | 20 | 1.31 (0.84; 2.04) | 0.242 | 11 | 1.25 (0.69; 2.27) | 0.460 |
| Leukaemia | 408994 | 1002 | 1.00 (Ref.) | 16 | 1.27 (0.77; 2.08) | 0.354 | 24 | 1.39 (0.92; 2.08) | 0.116 | 10 | 1.01 (0.54; 1.88) | 0.986 |

Data presented as adjusted hazard ratio (HR) and its 95% confidence interval (95% CI) by type of diets. Meat-eaters were used as the reference group. “Model 0” was unadjusted

"model 1" (minimally adjusted) included sociodemographic covariates (age, sex, deprivation, and ethnicity); "model 2" additionally included lifestyle factors (smoking, alcohol intake and total physical activity); "model 3" included model 2 plus multimorbidity: and “model 4” include model 3 plus body mass index.

Table S6: Characteristics of the cohorts included in systematic review; data *in italics* are those included in the meta-analysis.

| **Author (year)** | **Cancer site** | **Study cohort** | **Years Follow-up** | **Age range** | **Number of individuals** | **Number cases** | **Adjusted** |
| --- | --- | --- | --- | --- | --- | --- | --- |
| **Travis et al (2008)** | **Breast cancer** | **EPICOxford** | **7.4 years** | **20 - 89** | **37,643** | **585** | Height, body mass  index (BMI), age at menarche, age at first birth and parity, menopausal  status, current HRT use, alcohol consumption and daily  energy intake |
|  |  | **Number cases** | **Comparison** | **Results** |  |  |  |
|  |  |  |  | **HR** | **Lower CI** | **Upper CI** |  |
|  | *Breast* | *108* | *Vegetarian v/s Non-vegetarian* | *0.91* | *0.72* | *1.14* |  |
|  | *Premenopausal* | *55* | *Vegetarian v/s Non-vegetarian* | *0.95* | *0.68* | *1.32* |  |
|  | *Postmenopausal* | *33* | *Vegetarian v/s Non-vegetarian* | *0.79* | *0.54* | *1.16* |  |
| **Cade et al., (2010)** | **Breast cancer** | **UKWCS, (UK)** | **9 years** | **35-69** | **33,725** | **783** | Age, energy intake, menopausal status (combined analysis), calorie  adjusted fat, BMI, physical activity, OCP use, HRT use, smoking status, parity, age at  menarche, ethanol, total days breast feeding, socioeconomic class, level of education |
|  |  | **Number cases** | **Comparison** | **Results** |  |  |  |
|  |  |  |  | **HR** | **Lower CI** | **Upper CI** |  |
|  | *Breast* | *130* | *Vegetarian v/s Red meat eater* | *0.88* | *0.69* | *1.11* |  |
|  | *Breast* | *87* | *Pescatarian v/s Red meat eater* | *0.78* | *0.6* | *1.03* |  |
|  | *Premenopausal* | *83* | *Vegetarian v/s Red meat eater* | *0.92* | *0.67* | *1.24* |  |
|  | *Premenopausal* | *53* | *Pescatarian v/s Red meat eater* | *0.97* | *0.69* | *1.37* |  |
|  | *Postmenopausal* | *47* | *Vegetarian v/s Red meat eater* | *0.85* | *0.58* | *1.25* |  |
|  | *Postmenopausal* | *34* | *Pescatarian v/s Red meat eater* | *0.60* | *0.38* | *0.96* |  |
| **Tantamango-Bartley et al.,-2013** | **Overall cancer** | **Adventist Health Study-2.** | **4,14 years** | **30- 70** | **69,120** | **2939** | Race, family history of cancer, education, smoking, alcohol, age at menarche, pregnancies, breastfeeding, oral contraceptives, hormone replacement therapy, and menopause status**.** |
|  |  | **Number cases** | **Comparison** | **Results** |  |  |  |
|  |  |  |  | **HR** | **Lower CI** | **Upper CI** |  |
|  |  | *1526* | *Vegetarian v/s Non vegetarian* | *0.92* | *0.85* | *0.99* |  |
|  |  | *878* | *Lacto-vegetarian* | *0.93* | *0.85* | *1.02* |  |
|  |  | *276* | *Pescatarian v/s No vegetarian* | *0.88* | *0.77* | *1.01* |  |
| **Key TJ et al., (2014)** | **15 sites** | **OVS and EPIC–Oxford**  **Cohort, (UK)** | **14.9 years** | **20-89** | **61,647** |  | Smoking, alcohol consumption, physical activity level, BMI; + parity and oral contraceptive use for breast cancer  *corrected from misprinting in original paper |
|  |  | **Number cases** | **Comparison** | **Results** |  |  |  |
|  |  |  |  | **HR** | **Lower CI** | **Upper CI** |  |
|  | *Overall* | *1203* | *Vegetarian v/s No vegetarian* | *0.90* | *0.83** | *0.96* |  |
|  | *Overall* | *520* | *Pescatarian v/s No vegetarian* | *0.89* | *0.81* | *0.98* |  |
|  | Stomach | 11 | Vegetarian v/s No vegetarian | 0.38 | 0.2 | 0.71 |  |
|  | Stomach | 6 | Pescatarian v/s No vegetarian | 0.64 | 0.27 | 1.5 |  |
|  | Pancreas | 22 | Vegetarian v/s No vegetarian | 0.70 | 0.42 | 1.17 |  |
|  | Pancreas | 10 | Pescatarian v/s No vegetarian | 0.77 | 0.39 | 1.52 |  |
|  | Kidney | 21 | Vegetarian v/s No vegetarian | 1.02 | 0.58 | 1.78 |  |
|  | Kidney | 2 | Pescatarian v/s No vegetarian | 0.23 | 0.05 | 0.99 |  |
|  | Bladder | 24 | Vegetarian v/s No vegetarian | 0.65 | 0.40 | 1.03 |  |
|  | Bladder | 9 | Pescatarian v/s No vegetarian | 0.72 | 0.36 | 1.43 |  |
|  | *Colorectal* | *154* | *Vegetarian v/s No vegetarian* | *1.04* | *0.84* | *1.28* |  |
|  | *Colorectal* | *43* | *Pescatarian v/s No vegetarian* | *0.67* | *0.48* | *0.92* |  |
|  | *Colon* | *92* | *Vegetarian v/s No vegetarian* | *1.01* | *0.77* | *1.33* |  |
|  | *Colon* | *26* | *Pescatarian v/s No vegetarian* | *0.65* | *0.43* | *0.98* |  |
|  | *Rectum* | *62* | *Vegetarian v/s No vegetarian* | *1.08* | *0.79* | *1.48* |  |
|  | *Rectum* | *17* | *Pescatarian v/s No vegetarian* | *0.70* | *0.42* | *1.17* |  |
|  | *Lung* | *58* | *Vegetarian v/s No vegetarian* | *1.09* | *0.78* | *1.53* |  |
|  | *Lung* | *12* | *Pescatarian v/s No vegetarian* | *0.59* | *0.32* | *1.07* |  |
|  | *Prostate* | *100* | *Vegetarian v/s No vegetarian* | *0.83* | *0.64* | *1.06* |  |
|  | *Prostate* | *30* | *Pescatarian v/s No vegetarian* | *0.74* | *0.51* | *1.09* |  |
|  | *Breast* | *352* | *Vegetarian v/s No vegetarian* | *0.96* | *0.83* | *1.10* |  |
|  | *Breast* | *202* | *Pescatarian v/s No vegetarian* | *1.09* | *0.93* | *1.28* |  |
|  | Endometrium | 42 | Vegetarian v/s No vegetarian | 0.99 | 0.67 | 1.45 |  |
|  | Endometrium | 17 | Pescatarian v/s No vegetarian | 0.82 | 0.48 | 1.38 |  |
|  | Cervix | 27 | Vegetarian v/s No vegetarian | 1.90 | 1.00 | 3.60 |  |
|  | Cervix | 13 | Pescatarian v/s No vegetarian | 2.11 | 1.02 | 4.37 |  |
|  | Ovary | 56 | Vegetarian v/s No vegetarian | 0.87 | 0.61 | 1.22 |  |
|  | Ovary | 17 | Pescatarian v/s No vegetarian | 0.56 | 0.33 | 0.94 |  |
| **Gilsing et al., (2015)** | **Colorectal** | **NLCS-MIC,**  **(Netherlands)** | **20.3 years** | **55–69** | **10,210** | **437** | Age sex, total energy intake, cigarette smoking, alcohol consumption, BMI, non-occupational physical activity, and level of education  **Landmark data no show.** |
|  |  | **Number cases** | **Comparison** | **Results** |  |  |  |
|  |  |  |  | **HR** | **Lower CI** | **Upper CI** |  |
|  | *Colorectal* | *22* | *Vegetarian v/s No vegetarian* | *0.83* | *0.53* | *1.31* |  |
|  | *Colorectal* | *14* | *Pescatarian v/s Meat consumption group* | *0.88* | *0.51* | *1.51* |  |
|  | *Colon* | *19* | *Vegetarian v/s No vegetarian* | *1.01* | *0.62* | *1.66* |  |
|  | *Colon* | *11* | *Pescatarian v/s Meat consumption group* | *0.96* | *0.52* | *1.80* |  |
|  | *Rectum* | *1* | *Vegetarian v/s No vegetarian* | *0.21* | *0.03* | *1.55* |  |
|  | *Rectum* | *2* | *Pescatarian v/s Meat consumption group* | *0.68* | *0.16* | *2.84* |  |
| **Orlich et al., (2015)** | **Colorectal** | **AHS-2,**  **(USA and Canada)** | **7.3 years** | **≥25** | **77,659** | **490** | Age , race and sex, educational level, moderate or vigorous exercise, smoking, alcohol use , family history of colorectal cancer, history of peptic ulcer, history of inflammatory bowel disease, treatment for diabetes mellitus within the past year, used aspirin at least weekly at least 2 of the past 5 years, used statins at least 2 of the past 5 years, prior colonoscopy or flexible sigmoidoscopy , supplemental calcium use, supplemental vitamin D, dietary energy, and hormone therapy among menopausal women and body mass index |
|  |  | **Number cases** | **Comparison** | **Results** |  |  |  |
|  |  |  |  | **HR** | **Lower CI** | **Upper CI** |  |
|  | *Colorectal* | *252* | *Vegetarian v/s No vegetarian* | *0.79* | *0.64* | *0.97* |  |
|  | *Colorectal* | *35* | *Pescatarian v/s No vegetarian* | *0.58* | *0.40* | *0.84* |  |
|  | *Colorectal* | *147* | *Lactoovo v/s No vegetarian* | *0.83* | *0.66* | *1.05* |  |
|  | *Colon* | *197* | *Vegetarian v/s No vegetarian* | *0.83* | *0.66* | *1.05* |  |
|  | *Rectum* | *55* | *Vegetarian v/s No vegetarian* | *0.66* | *0.43* | *1.02* |  |
|  |  |  |  |  |  |  |  |
| **Gilsing et al., (2016)** | **Prostate**  **Breast and**  **Lung** | **NLCS-MIC, 20.3 years**  **(Netherlands)** |  | **55–69** | **11,082** |  | age, total energy intake, cigarette smoking, education.  **Landmark data no show** |
|  |  | **Number cases** | **Comparison** | **Results** |  |  |  |
|  |  |  |  | **HR** | **Lower CI** | **Upper CI** |  |
|  | *Prostate* | *19* | *Vegetarian v/s No vegetarian* | *1.09* | *0.68* | *1.76* |  |
|  | *Prostate* | *17* | *Pescatarian v/s No vegetarian* | *1.35* | *0.81* | *2.23* |  |
|  | *Breast* | *18* | *Vegetarian v/s No vegetarian* | *0.70* | *0.43* | *1.14* |  |
|  | *Breast* | *14* | *Pescatarian v/s No vegetarian* | *1.20* | *0.78* | *2.11* |  |
|  | *Lung* | *7* | *Vegetarian v/s No vegetarian* | *0.86* | *0.40* | *1.85* |  |
|  | *Lung* | *3* | *Pescatarian v/s No vegetarian* | *0.54* | *0.17* | *1.70* |  |
| **Tantamango-Bartley et al., -2016** | **Prostate** | **AHS-2, (USA**  **and Canada)** | **7.8 years** | **≥30** | **27,188** | **1079** | Race, family history of prostate cancer, education, screening for prostate cancer, energy intake and body mass index |
|  |  | **Number cases** | **Comparison** | **Results** |  |  |  |
|  |  |  |  | **HR** | **Lower CI** | **Upper CI** |  |
|  | Prostate | *333* | *Lacto-Vegetarian v/s No vegetarian* | *0.96* | *0.83* | *1.12* |  |
|  | Prostate | *121* | *Pescatarian v/s No vegetarian* | *1.07* | *0.88* | *1.31* |  |
| **Penniecook-Sawyers et al., 2016** | **Breast** | **AHS-2, (USA and Canada)** | **7.8 years** | **≥30** | **50,404** | **892** | Race, height, physical activity, family history of cancer, mammography in the last 2 years after age 42 years, age at menopause, age at menarche, birth control pills, hormone replacement therapy, age at first child, number of children, breastfeeding, educational level, smoking, alcohol, and body mass index |
|  |  | **Number cases** | **Comparison** | **Results** |  |  |  |
|  |  |  |  | **HR** | **Lower CI** | **Upper CI** |  |
|  | *Breast* | *478* | *Vegetarian v/s No vegetarian* | *1.00* | *0.87* | *1.16* |  |
|  | *Breast* | *289* | *Lacto-Vegetarian v/s No vegetarian* | *1.08* | *0.92* | *1.27* |  |
|  | *Breast* | *88* | *Pescatarian v/s No vegetarian* | *0.94* | *0.73* | *1.21* |  |
|  | *Premenopausal* | *83* | *Vegetarian v/s No vegetarian* | *1.14* | *0.81* | *1.61* |  |
|  | *Premenopausal* | *41* | *Lacto-Vegetarian v/s No vegetarian* | *0.98* | *0.64* | *1.48* |  |
|  | *Premenopausal* | *19* | *Pescatarian v/s No vegetarian* | *1.27* | *0.75* | *2.14* |  |
|  | *Postmenopausal* | *395* | *Vegetarian v/s No vegetarian* | *0.97* | *0.83* | *1.14* |  |
|  | *Postmenopausal* | *248* | *Lacto-Vegetarian v/s No vegetarian* | *1.10* | *0.92* | *1.31* |  |
|  | *Postmenopausal* | *69* | *Pescatarian v/s No vegetarian* | *0.88* | *0.66* | *1.18* |  |
| **Parra-Soto et al., (2021)** |  | **UK Biobank (UK)** | **8.8 years** | **37-73** | **409,110** |  | Age, sex, deprivation, and ethnicity, smoking, alcohol intake, total physical activity, multimorbidity and body mass index |
|  |  | **Number cases** | **Comparison** | **Results** |  |  |  |
|  |  |  |  | **HR** | **Lower CI** | **Upper CI** |  |
|  | *Overall* | *463* | *Vegetarian v/s No vegetarian* | *0.87* | *0.79* | *0.96* |  |
|  | *Overall* | *686* | *Pescatarian v/s No vegetarian* | *0.93* | *0.87* | *1.00* |  |
|  | *Colorectal* | *43* | *Vegetarian v/s No vegetarian* | *0.73* | *0.54* | *0.99* |  |
|  | *Colorectal* | *75* | *Pescatarian v/s No vegetarian* | *0.90* | *0.71* | *1.14* |  |
|  | *Colon* | *29* | *Vegetarian v/s No vegetarian* | *0.69* | *0.48* | *0.99* |  |
|  | *Colon* | *52* | *Pescatarian v/s No vegetarian* | *0.87* | *0.66* | *1.15* |  |
|  | *Rectum* | *18* | *Vegetarian v/s No vegetarian* | *0.72* | *0.45* | *1.15* |  |
|  | *Rectum* | *35* | *Pescatarian v/s No vegetarian* | *0.98* | *0.70* | *1.37* |  |
|  | *Lung* | *27* | *Vegetarian v/s No vegetarian* | *0.76* | *0.52* | *1.11* |  |
|  | *Lung* | *46* | *Pescatarian v/s No vegetarian* | *0.86* | *0.64* | *1.15* |  |
|  | *Prostate* | *47* | *Vegetarian v/s No vegetarian* | *0.57* | *0.43* | *0.76* |  |
|  | *Prostate* | *82* | *Pescatarian v/s No vegetarian* | *0.89* | *0.71* | *1.11* |  |
|  | *Breast* | *138* | *Vegetarian v/s No vegetarian* | *0.95* | *0.80* | *1.13* |  |
|  | *Breast* | *194* | *Pescatarian v/s No vegetarian* | *0.90* | *0.78* | *1.04* |  |
|  | *Premenopausal* | *50* | *Vegetarian v/s No vegetarian* | *1.00* | *0.75* | *1.33* |  |
|  | *Premenopausal* | *52* | *Pescatarian v/s No vegetarian* | *0.78* | *0.59* | *1.03* |  |
|  | *Postmenopausal* | *71* | *Vegetarian v/s No vegetarian* | *0.92* | *0.73* | *1.16* |  |
|  | *Postmenopausal* | *113* | *Pescatarian v/s No vegetarian* | *0.91* | *0.75* | *1.10* |  |

Table S7: Definitions of vegetarian, lacto-ovo-vegetarian, pescatarian and poultry diets used in the various studies.

|  | ***Vegetarian*** | ***Pescatarian*** | ***Lacto- Ovo-Vegetarian*** | ***Poultry*** |
| --- | --- | --- | --- | --- |
| **Travis et al., (2008)** | Did not eat meat, or fish | N/I | N/I | N/I |
| **Cade et al., (2010)** | Red meat, poultry, or fish less than once a week | Fish at least once a week but not poultry or red meat | N/I | Poultry at least once a week and can eat fish but not red meat |
| **Tantamango-Bartley et al.,**  **(2014)** | Red meat and poultry <1 per month | Red meat and poultry < 1 per  month, and fish ≥ 1 per month | Red meat, poultry and fish <1 per month, and eggs and dairy ≥ 1 per month. | N/I |
| **Key TJ et al., (2014)** | Did not eat meat, fish, eggs, or dairy products | Did not eat meat, but ate fish | N/I | N/I |
| **Gilsing et al., (2015)** | N/I | N/I | N/I | N/I |
| **Orlich et al., (2015)** | Red meat and poultry <1 per month | Fish one or more times a month, but all other meats less than once a month | Eggs / dairy 1 or more times a month, but fish and all other meats less than 1 time a month | N/I |
| **Gilsing et al., (2016)** | Do not eat meat or fish (including vegans, lacto-ovo-, lacto- and ovo-vegetarians) | Did not eat meat, but ate fish | N/I | N/I |
| **Tantamango-Bartley et al.,**  **(2016)** | Red meat, poultry, fish <1 time / month | Red meat or poultry <1 time / month, but fish ≥1 time / month and had no restrictions on the consumption of dairy products and / or eggs | Red meat, poultry and fish <1 time / month and eggs or dairy ≥1 time / month | N/I |
| **Penniecook-Sawyers et al.,**  **(2016)** | Do not eat meat or fish (including vegans, lacto-ovo-, lacto- and ovo-vegetarians) | Fish was ≥1 time a month, while red meat and poultry were consumed less than once a month, but no restrictions on dairy products or eggs | Fish, poultry and red meat were less than once a month and their intake of eggs or dairy products was greater than or equal to once a month | N/I |
| **Parra-Soto et al., (2021)** | Consumption of cheese and/or milk but not fish, poultry or red meat | Consumption of cheese, milk and fish but not poultry or red meat | N/I | Consumption of cheese, milk, fish and poultry but not red meat |

Table S8: Quality assessment of studies using a modified Newcastle-Ottawa scale for assessing studies in the systematic review of vegetarian diet and cancer risk.

| Study ID | Selection |  |  | Comparability* | Outcome |  | Total |
| --- | --- | --- | --- | --- | --- | --- | --- |
|  | Representativeness of exposed cohort (⋆) | Selection of non-exposed cohort (⋆) | Ascertainment of exposure (⋆) | (⋆⋆) | Assessment of outcome (⋆) | Adequacy of follow up (⋆) | **Total**  **(7⋆)** |
| Travis et al., (2008) | ⋆ | ⋆ |  | ⋆ | ⋆ | ⋆ | ⋆ ⋆ ⋆ ⋆ ⋆ (5) |
| Cade et al., (2010) | ⋆ | ⋆ |  | ⋆ | ⋆ | ⋆ | ⋆ ⋆ ⋆ ⋆ ⋆ (5) |
| Tantamango-Bartley et al., (2014) | ⋆ | ⋆ |  |  | ⋆ | ⋆ | ⋆ ⋆ ⋆ ⋆ (4) |
| Key TJ et al., (2014) | ⋆ | ⋆ |  | ⋆ | ⋆ | ⋆ | ⋆ ⋆ ⋆ ⋆ ⋆ (5) |
| Gilsing et al., (2015) |  |  |  | ⋆ | ⋆ | ⋆ | ⋆ ⋆ ⋆ (3) |
| Orlich et al., (2015) | ⋆ | ⋆ |  | ⋆ | ⋆ | ⋆ | ⋆ ⋆ ⋆ ⋆ ⋆ (5) |
| Gilsing et al., (2016) | ⋆ | ⋆ |  |  | ⋆ | ⋆ | ⋆ ⋆ ⋆ ⋆ (4) |
| Tantamango-Bartley et al., (2016) | ⋆ | ⋆ |  |  | ⋆ | ⋆ | ⋆ ⋆ ⋆ ⋆ (4) |
| Penniecook-Sawyers et al., (2016) | ⋆ | ⋆ |  | ⋆ | ⋆ | ⋆ | ⋆ ⋆ ⋆ ⋆ ⋆ (5) |
| Parra-Soto et al., (2021) | ⋆ | ⋆ |  | ⋆ ⋆ | ⋆ | ⋆ | ⋆ ⋆ ⋆ ⋆⋆ ⋆ (6) |

***** Comparability assessed as the following: one star rewarded if study excluded or adjusted for outcome, another star rewarded if study adjusted for age, race, smoking

Table S9: Risk of bias assessment (modified from Cochrane Tool to Assess Risk of Bias in Cohort Studies and EPOC Data Collection Form)

| **Study ID** | Allocation concealment (selection bias) | Assessment of exposure (self-report) | Outcome of interest present at beginning | Incomplete data | Selective reporting (reporting bias) | Total score* |
| --- | --- | --- | --- | --- | --- | --- |
| Travis et al., (2008) | + | - | + | + | + | 4 |
| Cade et al., (2010) | + | - | + | + | + | 4 |
| Tantamango-Bartley et al., (2014) | + | - | + | + | + | 4 |
| Key TJ et al., (2014) | + | - | + | + | + | 4 |
| Gilsing et al., (2015) | + | - | + | - | + | 3 |
| Orlich et al., (2015) | + | - | + | + | + | 4 |
| Gilsing et al., (2016) | + | - | + | + | + | 4 |
| Tantamango-Bartley et al., (2016) | + | - | + | + | + | 4 |
| Penniecook-Sawyers et al., (2016) | + | - | + | + | + | 4 |
| Parra-Soto et al., (2021) | + | - | + | + | + | 4 |

*Total score: points awarded based on number of “+” or low risk of bias

+ = Low risk of bias, ? = Unclear risk of bias, - = High risk of bias


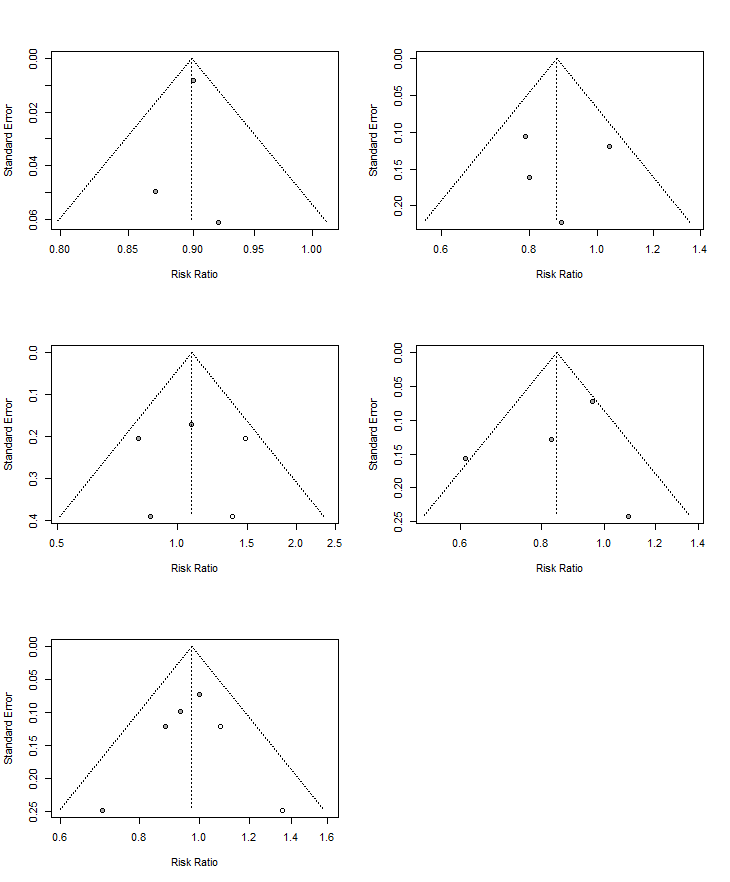


Fig. S2: Funnel plot of prospective cohort studies evaluating summary hazard ratios of colorectal, lung, prostate, breast and overall cancer for vegetarians versus meat-eaters (reference).


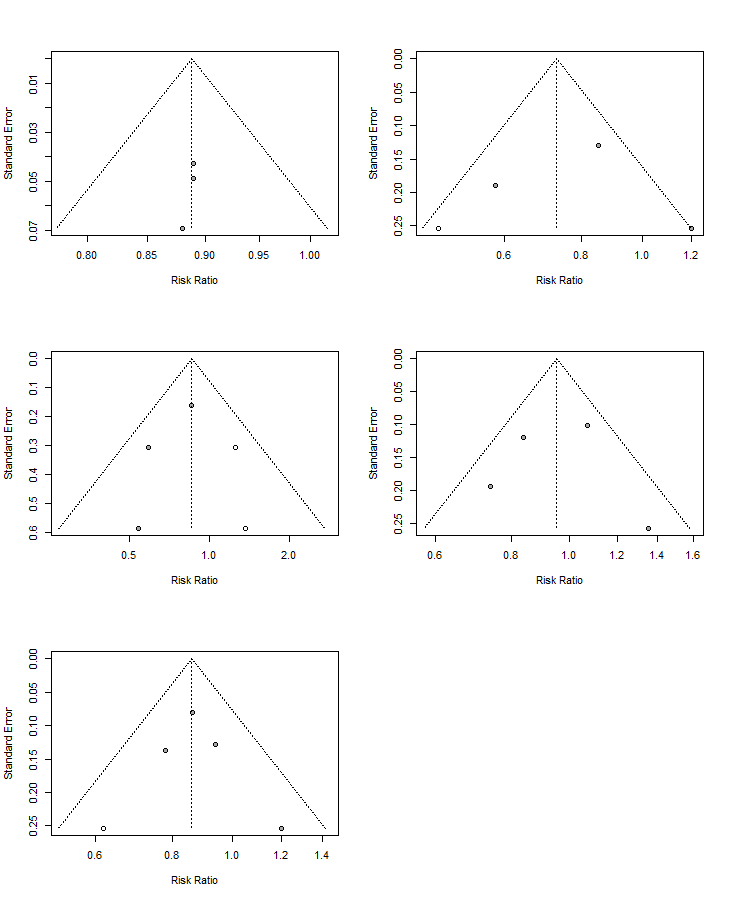


Fig.S3: Funnel plot of prospective cohort studies evaluating summary hazard ratios of colorectal, lung, prostate, breast and overall cancer for pescatarians versus meat-eaters (reference).


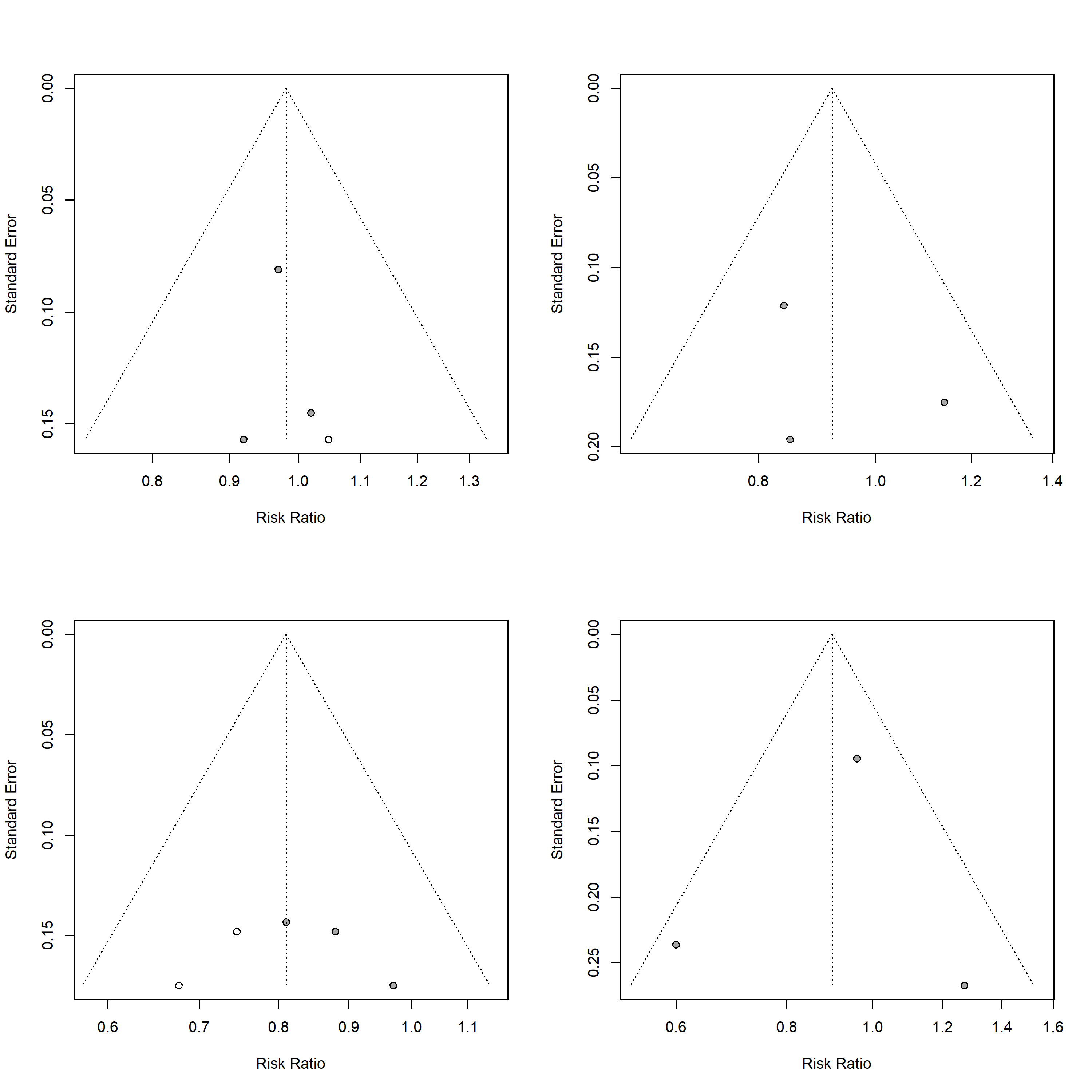


Fig S4: Funnel plot of prospective cohort studies evaluating summary hazard ratios of colon and rectum cancer for vegetarian and pescatarians versus meat-eaters (reference).


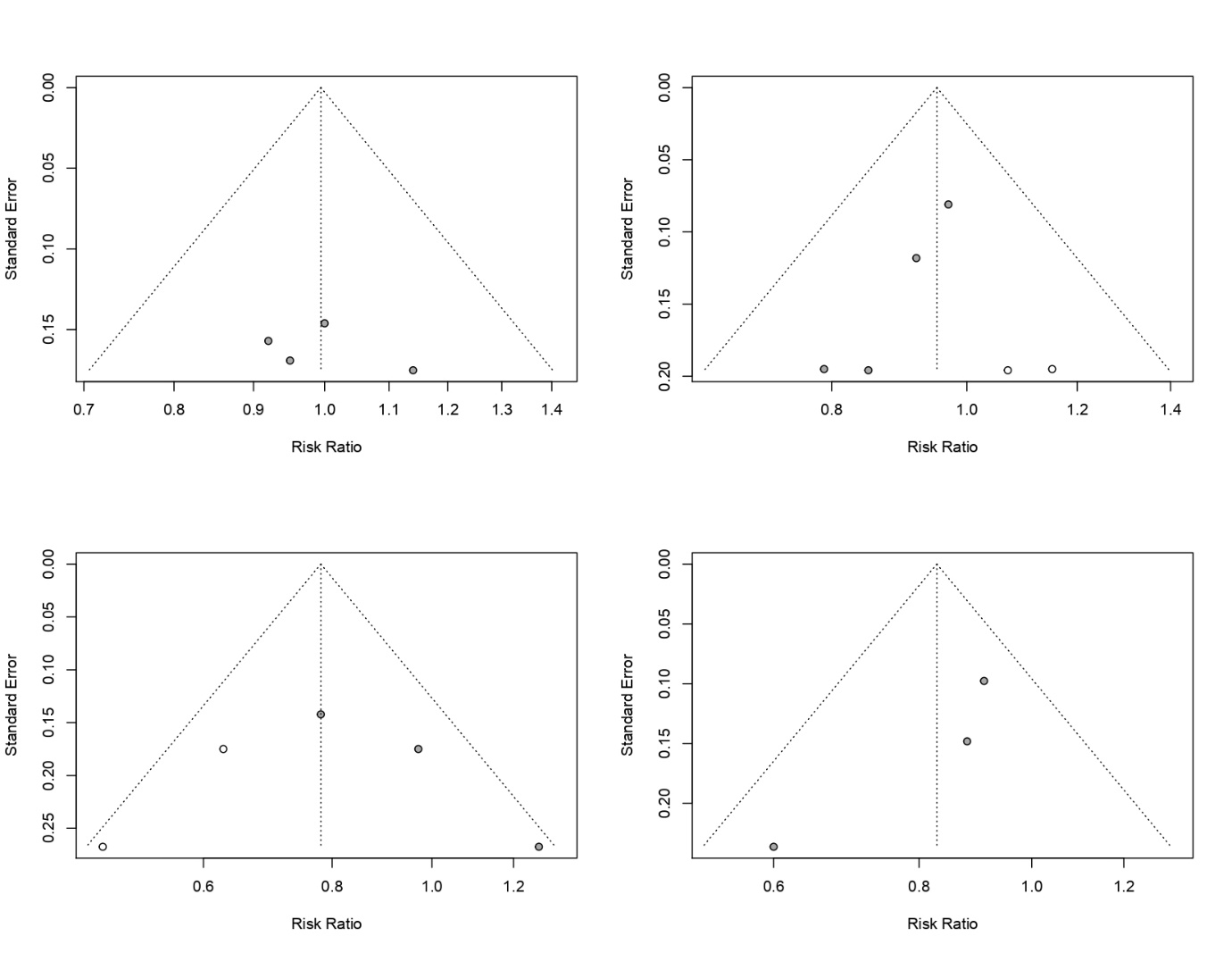


Fig S5: Funnel plot of prospective cohort studies evaluating summary hazard ratios of breast cancer in premenopausal and postmenopausal for vegetarian and pescatarians versus meat-eaters (reference).


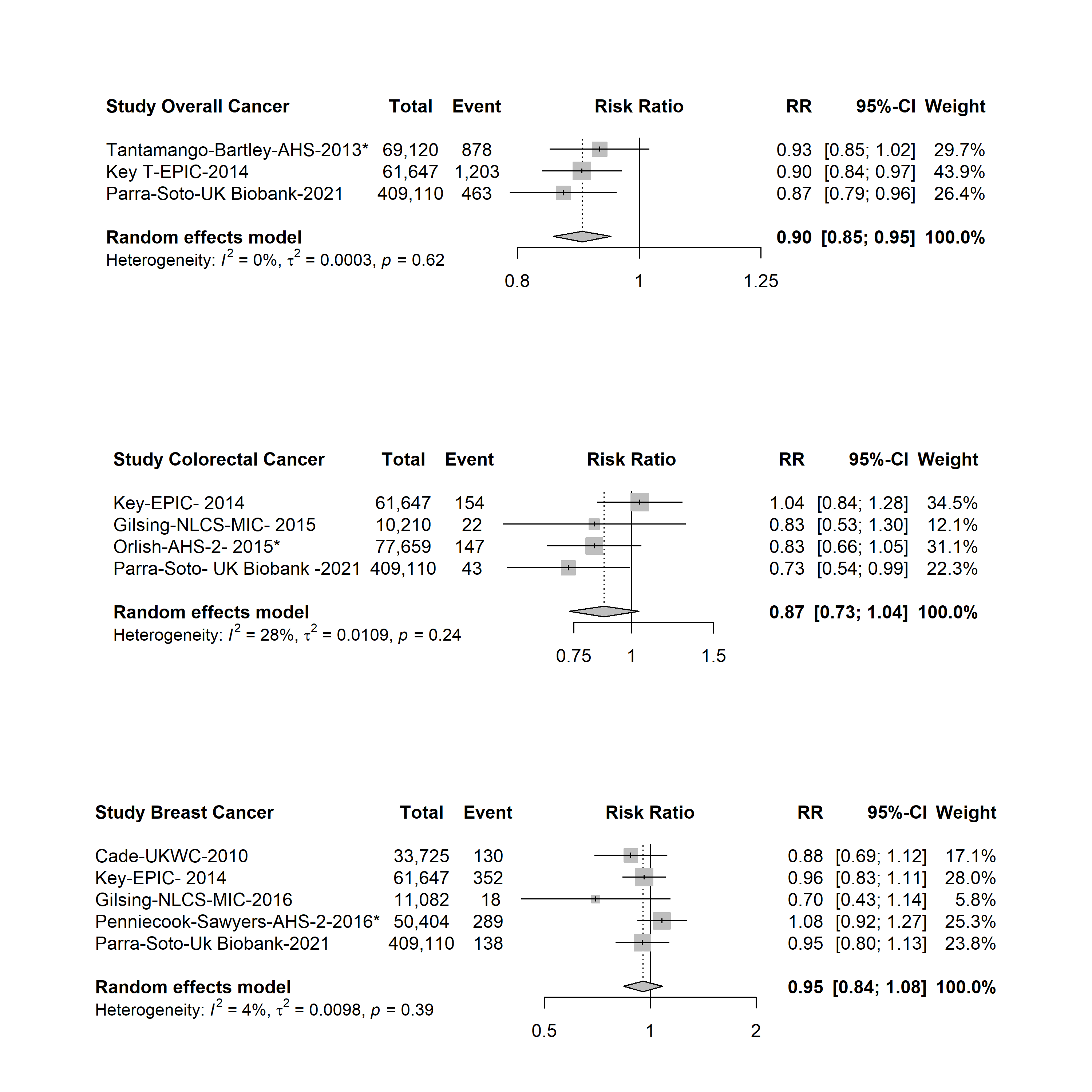


**Fig S6:** Sensitivity analysis of prospective cohort studies evaluating summary risk ratios of Lacto-Ovo vegetarians defined by Tantamango et al. for overall cancer, Orlish et al. for colorectal cancer, and Penniecook et al for breast cancer, compared with non-vegetarians (reference). RR: Risk ratio, CI: confidence interval.

*study change RR

**
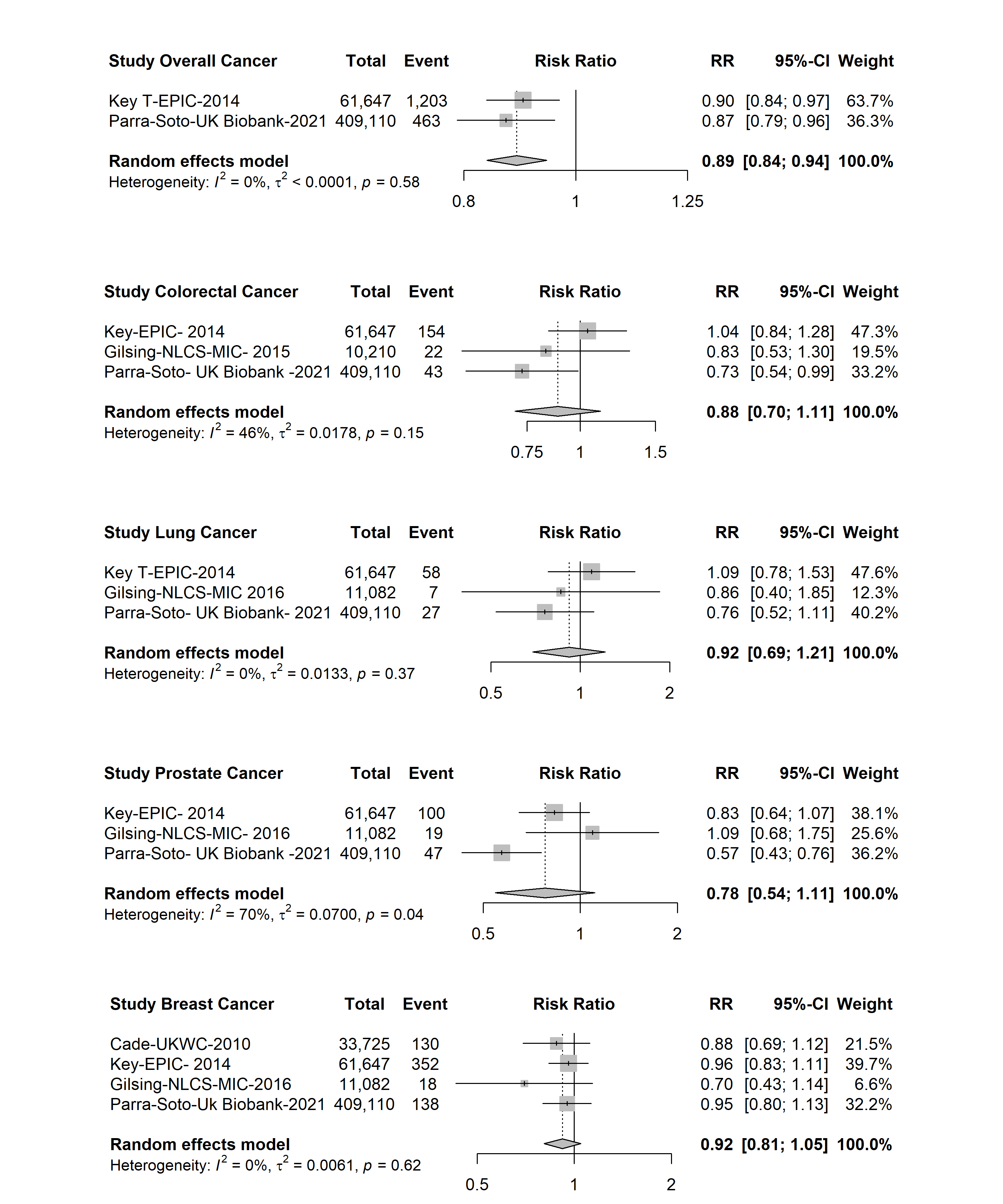
**

**Fig S7:** Sensitivity analysis of prospective cohort studies evaluating summary risk ratios of overall, colorectal, lung, prostate and breast cancer for vegetarians compared with meat-eaters (defined as reference). This sensitivity analysis excluded the study of Tantamango-Bartley et al., 2013 for overall cancer, Orlich et al 2015 for colorectal and Pennie-cook Sawyers et al., 2016 for breast cancer as their definition of vegetarians included a low intake of meat (less than once a month). RR: Risk ratio, CI: confidence interval

**
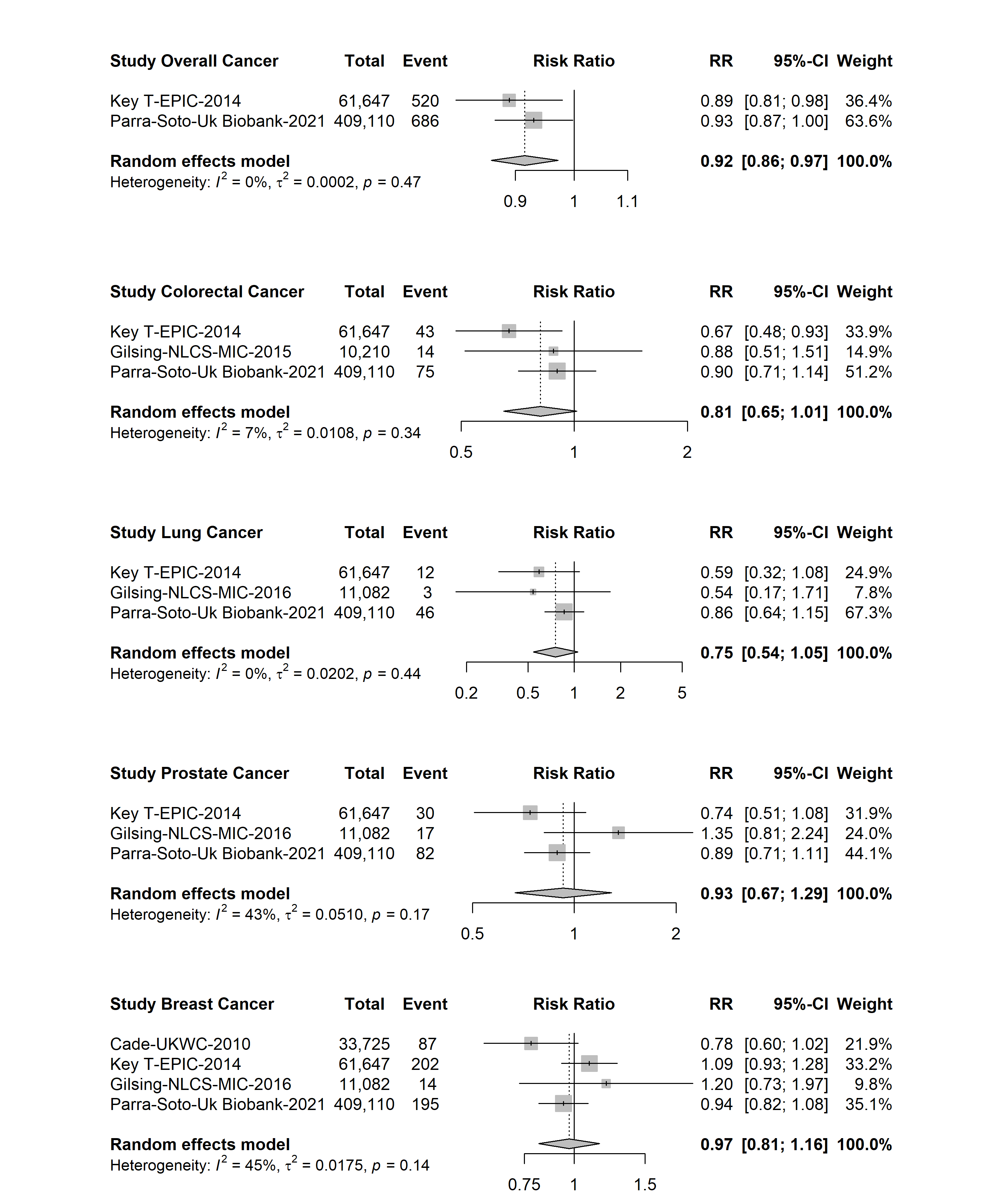
**

**Fig S8:** Sensitivity analysis of prospective cohort studies evaluating summary risk ratios of colorectal, lung, prostate, breast and overall cancer of pescatarians compared with meat-eaters (reference group). The studies of Tanta mango-Bartley et al., 2013 for overall cancer, Orlich et al 2015 for colorectal and Pennie-cook Sawyers et al., 2016 for breast cancer were removed as these included a definition of pescatarians who consumed meat less than once a month. RR: Risk ratio, CI: confidence interval

**
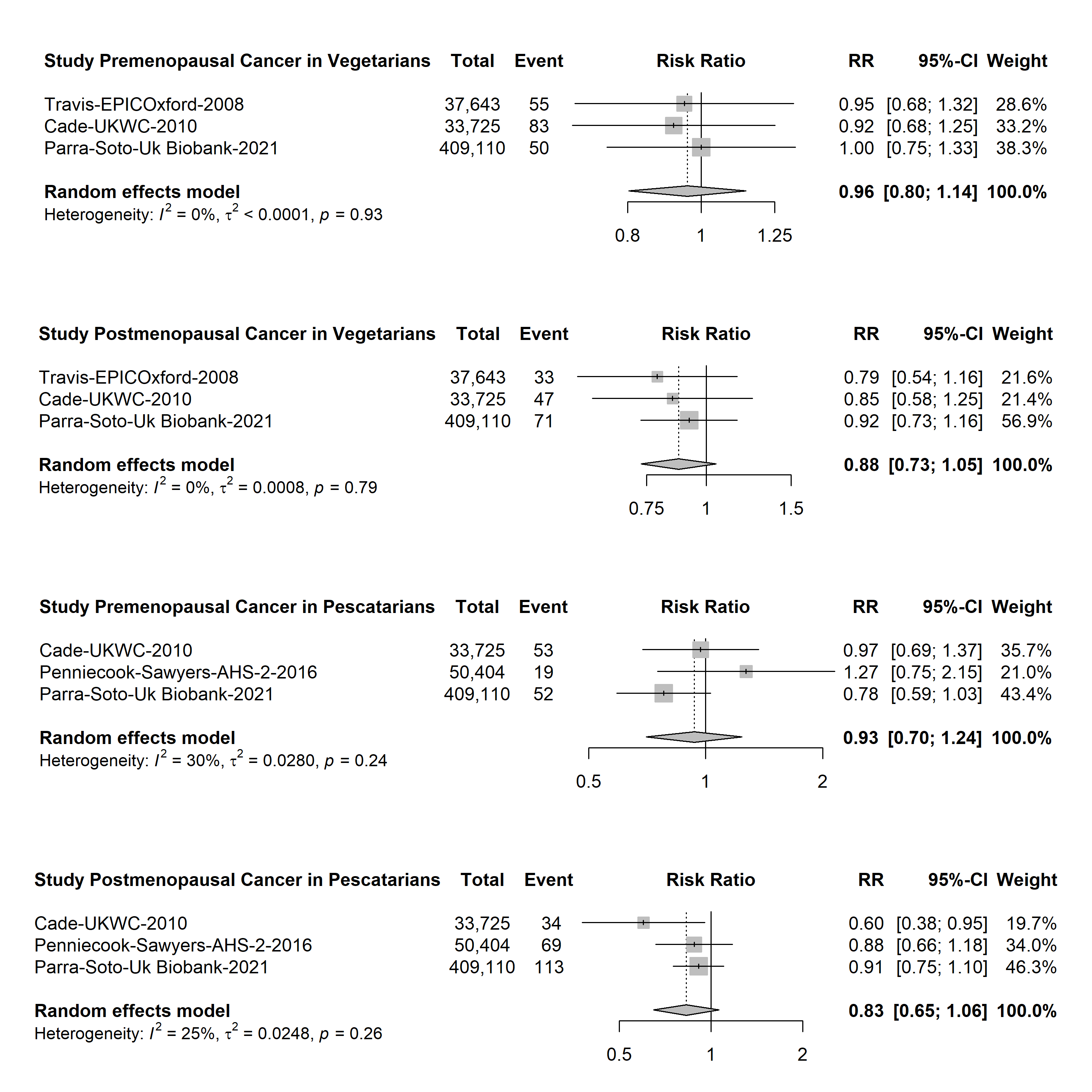
**

**Figure S9:** Forest plot of prospective cohort studies evaluating summary risk ratios of breast cancer in premenopausal and postmenopausal women for vegetarians and pescatarians compared with meat-eaters (reference). RR: Risk ratio, CI: confidence interval. Removing Pennie-cook Sawyers et al., 2016
